# Exploring the structures, stability, and light absorption properties of three thiostannates synthesised at similar conditions

**DOI:** 10.1038/s41598-021-01329-9

**Published:** 2021-11-11

**Authors:** Henrik S. Jeppesen, Peter Nørby, Jens Jakob Gammelgaard, Kasper Borup, Nina Lock

**Affiliations:** 1 Interdisciplinary Nanoscience Center (iNANO), Aarhus University, Gustav Wieds Vej 14, 8000 Aarhus C, Denmark; 2 Sino-Danish Center for Research and Education (SDC), Beijing, China; 3 Department of Chemistry, Center for Materials Crystallography (CMC), Aarhus University, Langelandsgade 140, 8000 Aarhus C, Denmark; 4 Department of Biological and Chemical Engineering, Carbon Dioxide Activation Center (CADIAC), Aarhus University, Åbogade 40, 8200 Aarhus N, Denmark

**Keywords:** Chemistry, Materials science

## Abstract

We present the synthesis, crystal structures and optical properties of three thiostannates prepared by using 1-(2-aminoethyl)piperazine (AEPz) as structure directing agent. Two of the thiostannates are layered materials (AEPz-SnS-1 and AEPz:EtOH-SnS-1) consisting of [Sn_3_S_7_^2−^]_n_ sheets with organic cations located in-between. The third compound is a molecular thiostannate (Sn_2_S_6_(AEPzH_2_)_2_) composed of dimeric Sn_2_S_6_^4−^ and AEPzH_2_^2+^. In preparation of the layered compounds, the use of AEPz as the only solvent results in AEPz-SnS-1 with regular hexagonal pores and crystallographically disordered organic cations. In contrast, a mixture of AEPz and absolute ethanol gives AEPz:EtOH-SnS-1 with distorted hexagonal pores and ordered cations between the layers. The influence of cation order on the light absorption properties and the material thermal stability was investigated through thermal treatment of the layered compounds up to 200 °C. Both compounds show colour changes when heated, but cation order results in larger thermal stability. For AEPz-SnS-1, a decreased inter-layer distance and substantial loss of organic matter was observed when heated. However, pair distribution function analysis reveals that the local in-layer thiostannate structure of AEPz-SnS-1 remains unchanged. In contrast, AEPz:EtOH-SnS-1 does not undergo noticeable structural changes by the thermal treatment. All materials are optical semiconductors with band gaps of 3.0–3.1 eV.

## Introduction

A range of properties including photocatalysis has been reported for structurally complex metal sulfides^1–5^. For example, tin sulfides and thiostannates comprise a family of structurally diverse compounds, and layered SnS_2_ has been studied thoroughly due to its photocatalytic properties with^6,7^ and without dopant modifications^8,9^. Multiple thiostannates have been reported, including molecular compounds containing the Sn_2_S_6_^4−^ ion^10–20^, polymeric layered materials containing [Sn_3_S_7_^2−^]_n_ or [Sn_4_S_9_^2−^]_n_ sheets^13,21–25^, and three-dimensional compounds containing [Sn_2_S_5_^2−^]_n_^26^. The negative charge is balanced by molecular organic cations such as alkylammonium ions^24,25^, protonated amines^12,13,21,23^, or by metal ions or metal complexes^10,11,13–16^. The thiostannates are optical semiconductors; the molecular compounds with metal-free cations have band gaps in the range ~ 3.5–4.2 eV^27,28^, while band gaps of  ~ 2.5–3.2 eV have been reported for the layered materials^23,24^. Hence, the properties of thiostannates might be tailored due to their structural diversity and optical properties.

A range of properties has been reported for members of the R_2_Sn_3_S_7_ family of layered materials (R is a cation), which are also denoted R-SnS-1^29^. Some alkylammonium containing R-SnS-1 type compounds were reported to be photocatalytically active for dye degradation using visible light^24^. Other R-SnS-1 compounds have shown ion exchange properties, allowing for modification of their light absorption properties^30–32^. Recently, we observed that some R-SnS-1 compounds retain their optical properties, despite undergoing a crystalline to amorphous phase transition in water, while conserving their local structure^33^.

**Figure 1:**
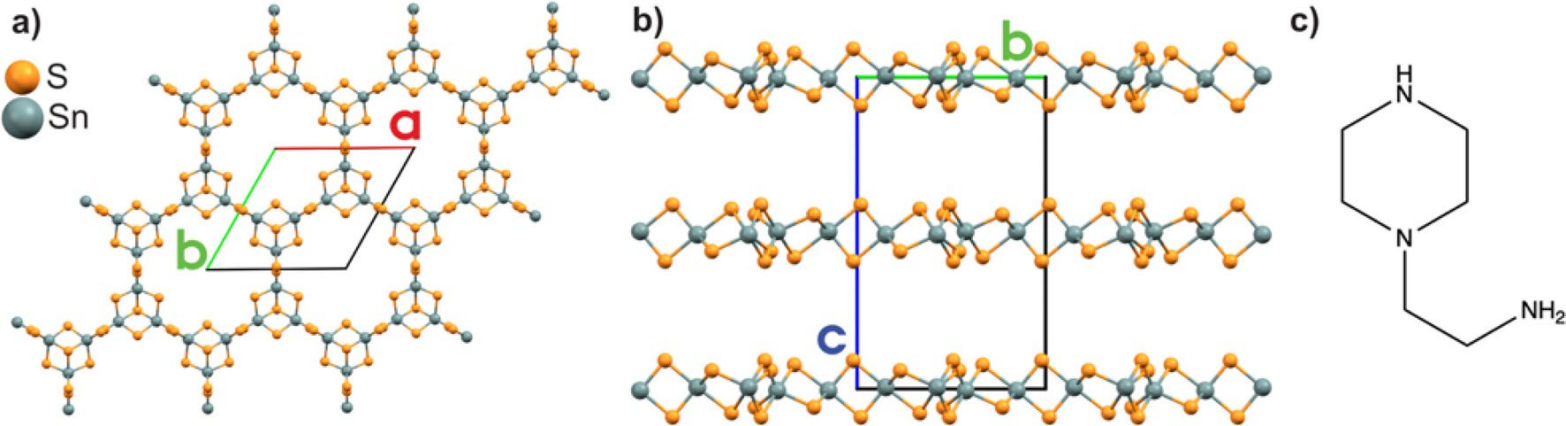
(**a**) Top view of the AEPz-SnS-1 structure with regular hexagonal pores, and (**b**) Side view of ABAB stacking layers of AEPz-SnS-1. (**c**) Molecular structure of AEPz used as structure directing agent and solvent.

The crystal structure and compound stability are of key importance in relation to understanding the properties and potential of R-SnS-1 type materials. The fundamental building unit of the [Sn_3_S_7_^2−^]_n_ layer in R-SnS-1 is the Sn_3_S_4_^4+^ broken-cube cluster, which are connected by double sulfur bridges to create a 24-atom hexagonally shaped pore (Fig. 1a). The charge compensating ions are located between the layers (Fig. 1b). The cations are either crystallographic ordered, such as in DABCOH-SnS-1 (DABCO = 1,4-Diazabicyclo[2.2.2]octane)^21^ and AEP-SnS-1 (AEP = 1-(2-aminoethyl)piperidine)^23^, or disordered as observed in e.g. trenH-SnS-1 (tren = tris(2-aminoethyl) amine)^13,23^. Depending on the structure directing agent used in the solvothermal synthesis (e.g. amines), the pores are either regular or distorted hexagons, and the inter-layer separation spans widely from e.g. 7.40 Å in FJSM-SnS-1 ([Me_2_NH_2_]_4/3_[Me_3_NH]_2/3_Sn_3_S_7_)^31^ to 9.52 Å in trenH-SnS-1^13,23^.

The formation mechanism of R-SnS-1 type compounds has been reported to occur via a polymerization process in which a cationic species R is used as structure directing agent under solvothermal conditions. Initially, a dimeric Sn_2_S_6_^4−^ based intermediate is formed, which subsequently combine to form polymeric honeycomb-like monolayers with the composition [Sn_3_S_7_^2−^]_n_. Lastly, these layers stack to give a layered compound with intercalated of cations R in-between^34,35^. While R-SnS-1 is considered the final product, the formation of the layered compound has previously been haltered and allowed isolation of crystals of the corresponding dimeric compound R_2_Sn_2_S_6_. As an example, polymeric trenH-SnS-1^13,23^ and molecular Sn_2_S_6_(trenH_2_)_2_^17^ have been synthesised selectively at different conditions by using the same amine.

To tailor materials towards specific applications, it is crucial to obtain synthesis control allowing preparation of products with desired properties. Even though several R-SnS-1 type compounds have been reported, many unanswered questions remain with respect to the role of the structure directing agent on the resulting structure in terms of e.g. cation order/disorder and regular versus distorted hexagonal pores. These structural properties might influence the material band gap and the compound stability.

In recent publications, we have presented the synthesis of thiostannates using 1-(2-aminoethyl)piperazine (AEPz, Fig. 1c) as solvent and structure directing agent^33,36^, but without focusing on the crystal structures in detail. Instead, in the study by Walther et al.^36^ enzyme-like activity was demonstrated for the compounds, and only the fundamental structure was reported for layered AEPz:EtOH-SnS-1 and molecular Sn_2_S_6_(AEPzH_2_) without in-depth studies of e.g. cation–anion interactions and crystal packing. Finally, AEPz-SnS-1 was proposed to be isostructural to trenH-SnS-1 based on X-ray diffraction data^33,36^. In this paper, we present optimized synthesis protocols giving molecular Sn_2_S_6_(AEPzH_2_)_2_ and two R-SnS-1 type compounds (AEPz-SnS-1 and AEPz:EtOH-SnS-1) by simple variation of stoichiometry, solvent, and temperature. Moreover, the crystal structures of the three thiostannates are presented in detail. Finally, as the same structure directly amine (AEPz) uniquely gives two different layered R-SnS-1 compounds with ordered and disordered cations, respectively, the influence of cation order on some material properties were studied. This was done by investigating the light absorption properties and thermal stability of the compounds by diffuse reflectance spectroscopy (DRS), PXRD and X-ray total scattering and pair distribution function (PDF) analysis.

## Results and discussion

**Figure 2:**
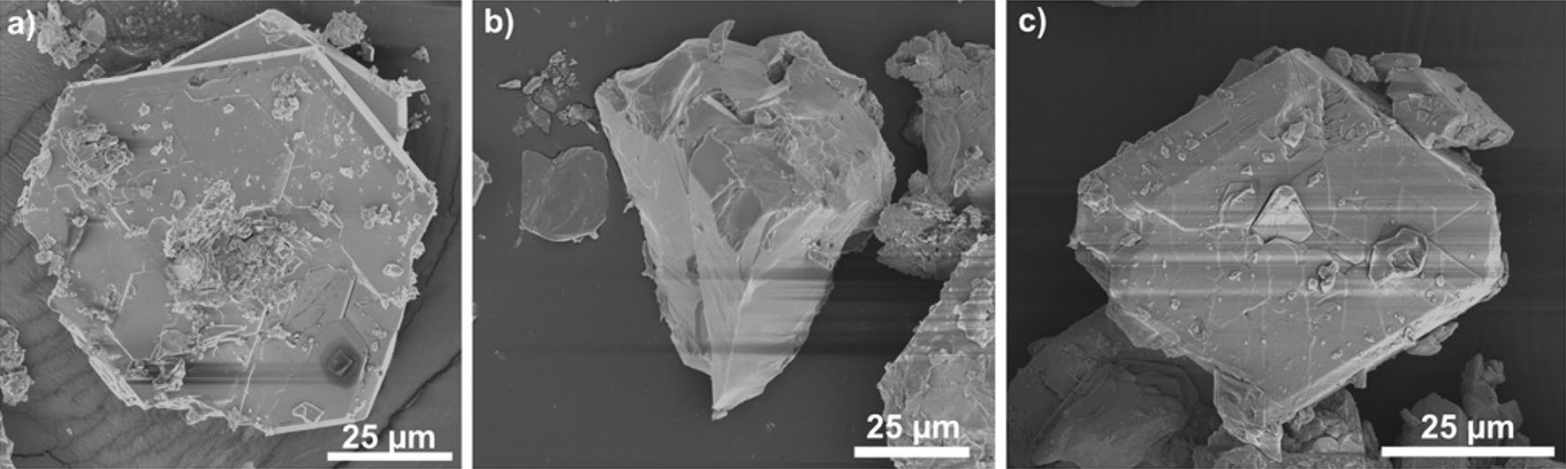
Scanning electron microscopy (SEM) images of (**a**) hexagonal AEPz-SnS-1, (**b**) irregularly shaped crystals of Sn_2_S_6_(AEPzH_2_)_2_ and (**c**) rhombic AEPz:EtOH-SnS-1. Low magnification SEM images can be found in Fig. S2, S5, and S10.

**Figure 3:**
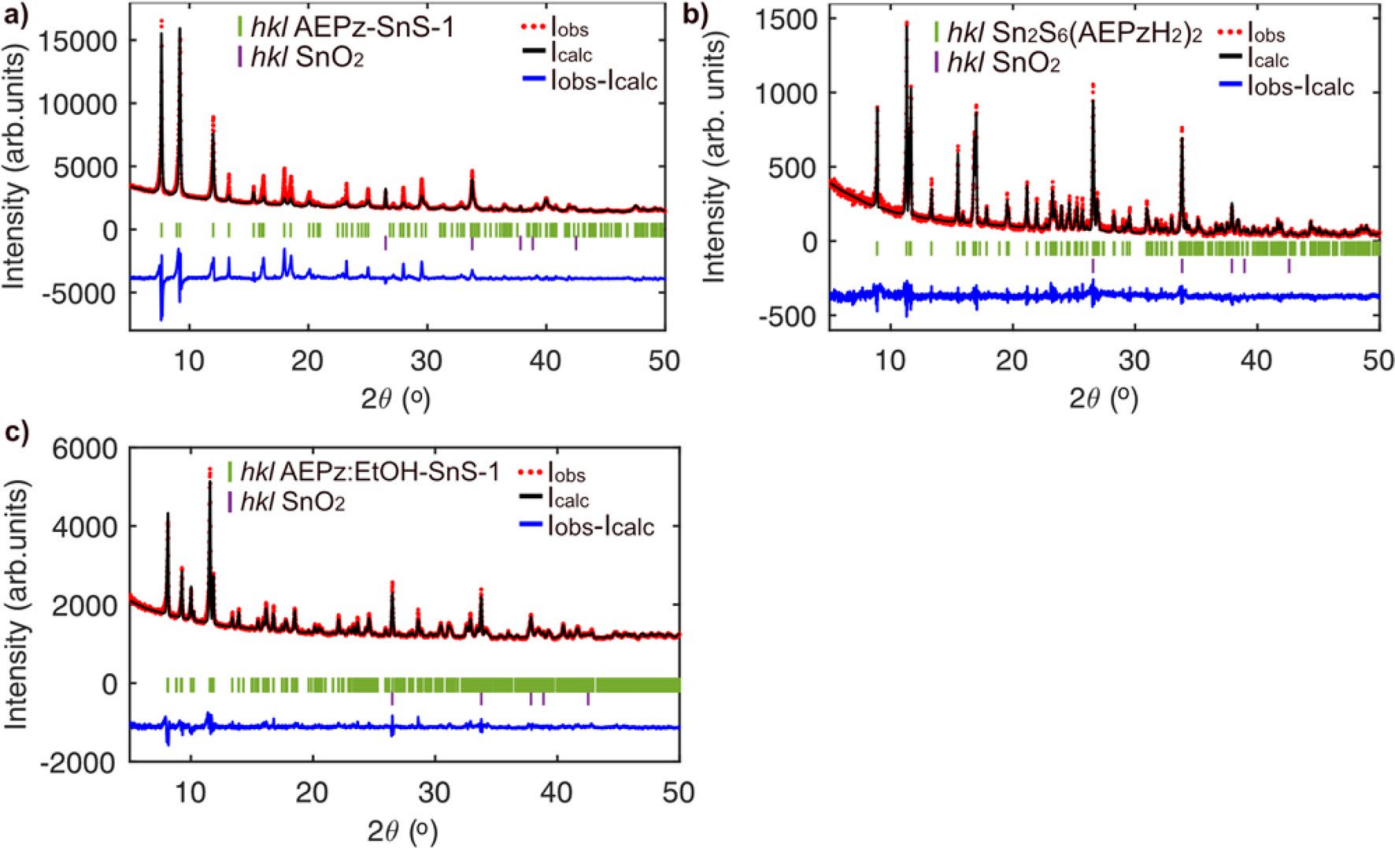
Rietveld refinement of PXRD data for (**a**) AEPz-SnS-1, (**b**) Sn_2_S_6_(AEPzH_2_)_2_, and (**c**) AEPz:EtOH-SnS-1. Structural models for the thiostannates were obtained from structure solutions based on single crystal diffraction (this paper), while CIF data for SnO_2_ was obtained from ICSD entry 9163^37^. See refinement details in Table S2, S3 and S8.

Three different thiostannates were synthesised selectively as a result of stoichiometry, solvent and temperature variation. By heating the precursors in a SnO_2_:S molar ratio of 3:8 in AEPz at 190 °C (and potentially applying a cooling ramp for improved crystal quality, see the Methods section), a pale green powder containing flat hexagonal crystals of AEPz-SnS-1 (Fig. 2a) was obtained. By lowering the temperature and changing the molar ratio of SnO_2_:S to approx. 1:10, dimeric Sn_2_S_6_(AEPzH_2_)_2_ was obtained as irregularly shaped transparent reddish crystals (Fig. 2b). By using a preparation method similarly to that of AEPz-SnS-1, but by adding ethanol as co-solvent to the reaction mixture, small green rhombic crystals of AEPz:EtOH-SnS-1 (Fig. 2c) were obtained along with a powder. The three thiostannates can be synthesized selectively, but the resulting powders contain remains of precursor SnO_2_. According to Rietveld refinements, we estimate the mass fraction of SnO_2_ to be ~ 12% in Sn_2_S_6_(AEPzH_2_)_2_, ~ 8% in AEPz-SnS-1, and ~ 11% in AEPz:EtOH-SnS-1 (Fig. 3a–c and S4, S7, S14, and Table S2, S3 and S8). Details on the refinements are presented in the ESI, and calculated patterns based on CIF data from single crystal diffraction are shown in Fig. S3, S6, and S11.

By conducting the synthesis under conditions deviating slightly from those reported, a mixture of different thiostannates would typically result (see e.g., Fig. S1 and S8). Tuning the synthesis conditions for the thiostannates to fully avoid SnO_2_, would therefore compromise the selectivity with respect to the thiostannate phase. However, AEPz-SnS-1 has previously been obtained as a phase pure powder via a two-step procedure at the cost of crystal quality, due to grinding of the powder after the first step^33^.

### Structure solution by single crystal diffraction

The crystal structures of the three thiostannates were solved by single crystal X-ray diffraction. A summary of the crystallographic details is shown in Table 1. Due to the layered nature of the R-SnS-1 compounds, synthesising high quality crystals is known to be problematic^38^. As a result, the obtained R-SnS-1 crystals are only of moderate quality (see ESI for a detailed explanation)**.** Based on the proposed chemical formulas (Table 1, entry 2), the material densities have been calculated. The material with the lowest density is Sn_2_S_6_(AEPzH_2_)_2_ at 1.83 g·cm^−1^, while the R-SnS-1 compounds have increased densities of 1.98 and 2.21 g·cm^−1^, respectively, for AEPz-SnS-1 and AEPz:EtOH-SnS-1. The latter indicates a more compact crystal packing of AEPz:EtOH-SnS-1 in comparison with AEPz-SnS-1.

**Table 1:** Crystallographic data for the three thiostannates.

Compound	AEPz-SnS-1	Sn_2_S_6_(AEPzH_2_)_2_	AEPz:EtOH-SnS-1
Refined chemical formula	Sn_3_S_7_	SnS_3_·C_6_N_3_H_17_	Sn_6_S_14_·C_16_N_8_
Expected chemical formula	Sn_3_S_7_·C_12_N_6_H_32_	SnS_3_·C_6_N_3_H_17_	Sn_6_S_14_·C_16_N_8_H_42_
*M*_r_ (u)	580.49	346.09	1465.22
*T* (K)	300	100	300
Crystal system	Hexagonal	Triclinic	Triclinic
Space Group	*P*6_3_/*mmc*	*P* $\overline{1 }$	*P* $\overline{1 }$
*a* (Å)	13.135(5)	7.6079(6)	11.848(2)
*b* (Å)	13.135(5)	8.4117(6)	11.851(2)
*c* (Å)	18.849(6)	10.7128(7)	18.233(3)
*α* (°)	90	110.954(2)	76.19(2)
*β* (°)	90	98.482(2)	76.24(2)
*γ* (°)	120	92.676(2)	67.46(2)
*V* (Å^3^)	2816(2)	629.55(8)	2264.0(7)
*Z*	4	2	2
*ρ*/(g/cm^3^) (diffraction)	1.369	1.826	2.149
*F*(000)	1048	344	1352
*θ*_min_, *θ*_max_ (°)	3.6, 15.9	2.46, 30.57	3.6, 19.6
*µ* (mm^−1^)	3.13	1.31	3.93
Collected reflections	8478	51,265	39,170
Unique reflections (all)	287	7189	6495
Unique reflections [*I* ≥ 2*σ*(*I*)]	193	6693	3106
Parameters/restraints	22/ 0	186/ 0	326/ 61
*R*1 (all data), *R*1-factor[*I* ≥ 2*σ*(*I*)] (%)	12.0, 9.85	2.56, 2.18	17.6, 7.69
Goodness-of-fit	1.464	1.168	1.001
Δ*ρ*(max, min) (e/Å^3^)	1.70, –1.32	1.985, –1.038	1.315, –0.973

The Refined chemical formula (entry 1) refers to the composition as determined by single crystal diffraction, whereas the Expected chemical formula (entry 2) gives the materials composition based on combined single crystal diffraction analysis and elemental analysis/hydrogen balance.

### Crystal structure of AEPz-SnS-1

Hexagonal plates of AEPz-SnS-1 with a width of approx. 50 µm or less and a thickness of approx. 20 µm (Fig. 2a) were obtained by applying a 30-hour cooling ramp during synthesis (see Methods). Using this procedure gave an impurity of Sn_2_S_6_(AEPzH_2_)_2_ (Fig. S1), but also AEPz-SnS-1 crystals of a higher, however not ideal, quality for single crystal diffraction.

The structure of the thiostannate layers in AEPz-SnS-1 was solved in the hexagonal space group *P*6_3_/*mmc* (no. 194) (Table 1, Fig. 1). The layers are stacked in an ABAB sequence along the [001] direction with an inter-layer distance of 9.425(6) Å. Tin has trigonal bipyramidal coordination with Sn–S bond lengths between 2.37(2) and 2.603(2) Å and S–Sn–S’ angles between 87.9(5)° and 176.9(7)°. A full list of bond distances and angles can be found in Table S1. In AEPz-SnS-1, the [Sn_3_S_7_^2−^]_n_ layers have regular hexagonal pores with a diameter of 13.13(4) Å between opposite bridging sulfur atoms. The molecular cations were found to be crystallographically disordered, and the large interlayer spacing between the thiostannate layers suggests the cations to be located between these. We previously reported that the compound contains two protonated AEPz (AEPzH^+^) per [Sn_3_S_7_^2−^] moiety according to CHNS analysis^33^. Herein, by using the SQUEEZE procedure^39^, 102 electrons were removed per [Sn_3_S_7_^2−^], equivalent of approx. 1.5 AEPz (110 electrons). Due to the moderate crystal quality (see ESI), we suggest two AEPzH^+^ per [Sn_3_S_7_^2−^], leading to a charge neutral compound. Thus, we can conclude that the thiostannate sheets of trenH-SnS-1^13,23^ and AEPz-SnS-1 are isostructural as previously proposed^33,36^ despite of using structure directing organic amines with substantially different size and shape.

### Crystal structure of Sn_2_S_6_(AEPzH_2_)_2_

**Figure 4:**
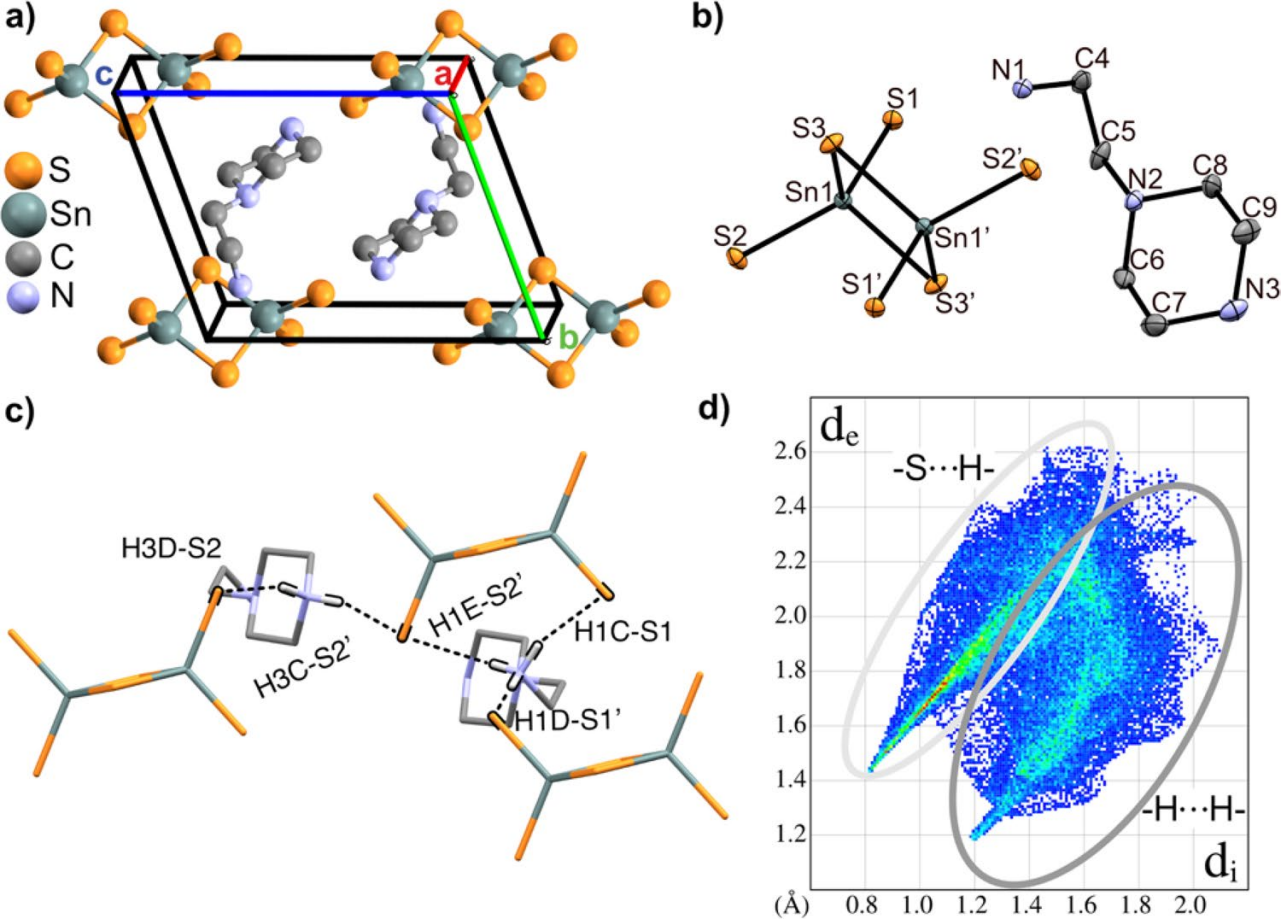
(**a**) Crystal structure of Sn_2_S_6_(AEPzH_2_)_2_ solved in *P* $\overline{1 }$. (**b**) ORTEP representation of the Sn_2_S_6_^4−^ and AEPzH_2_^2+^ ions (50% probability ellipsoids). Hydrogen atoms have been omitted for clarity in (**a**,**b**). (**c**) Hydrogen bonding between AEPzH_2_^2+^ and Sn_2_S_6_^4−^. All hydrogen atoms except for H1C-E bound to N1 and H3C-D bound to N3 (shown in white) have been omitted. Hydrogen bonds are displayed as dotted lines and black caps around the atoms of interest. (**d**) Two-dimensional fingerprint plot based on Hirshfeld surface analysis of AEPzH_2_^2+^ in the Sn_2_S_6_(AEPzH_2_)_2_ structure showing the closest contacts are cation-cation –H $\cdots$ H– and cation–anion –H $\cdots$ S– interactions. Further details are given in the Methods section.

By using an SnO_2_:S precursor ratio of 1:10 and a temperature of 150 °C, formation of the polymeric sheets can be haltered to give crystals of dimeric Sn_2_S_6_(AEPzH_2_)_2_. The synthesis of Sn_2_S_6_(AEPzH_2_)_2_ mainly gives irregularly shaped crystals in various sizes, of which a representative example is shown in Fig. 2b. The structure was solved in the triclinic space group *P* $\overline{1 }$ (no. 2) and is composed of Sn_2_S_6_^4−^ ions (two edge sharing tetrahedra) and charge stabilising ammonium ions (Fig. 4a,b). The positions of all hydrogen atoms were determined from the electron density map and refined without any restraints. Notably, both the primary (N1) and secondary (N3) amine functionalities are protonated to form AEPzH_2_^2+^.

All bond lengths and angles are shown in Table S5. The Sn-S bond lengths are in the range 2.3292(3) Å-2.4454(3) Å, and the S-Sn-S’ angles vary between 91.85(1)^o^ and 114.44(1)^o^ and thereby deviate substantially from the ideal tetrahedron. Finally, the Sn-S-Sn’ angle across the sulfur bridge is found to be 88.15(1)^o^. Overall, the bond distances and angles are in good agreement with previously reported R_2_Sn_2_S_6_ compounds^12,18^. The lengths of the –N–H $\cdots$ S– hydrogen bonds span a relatively large interval of 2.29(3) Å–2.64(3) Å (Table S4 and Fig. 4c) and are comparable to those reported for Sn_2_S_6_(trenH_2_)_2_^18^. The protonated primary (N1) and secondary (N3) amino groups show strong interactions with neighbouring Sn_2_S_6_^4−^ clusters, by each interacting with two thiostannate dimers (Fig. 4c). The tertiary amine (N2) is interacting less with the Sn_2_S_6_^4−^ clusters, which is seen by the closest –N2 $\cdots$ S3– contact being 4.030(1) Å, i.e. a distance which is approx. 15% longer than the corresponding distances involving –N1 $\cdots$ S1– and –N3 $\cdots$ S2–. Instead, the tertiary amine (N2) shows a stronger interaction with the ethylamine moiety of AEPzH_2_^2+^ with a hydrogen bonding distance (N2 $\cdots$ H1E-N1) of 2.67(3) Å. It is meaningful that the lone pair of N2 interacts with the cationic species, while the ammonium groups (N1 and N3) interact with Sn_2_S_6_^4−^.

A Hirshfeld surface of the AEPzH_2_^2+^ molecule in Sn_2_S_6_(AEPzH_2_)_2_ was calculated. Overall, there is good agreement between the qualitative analysis of the hydrogen bonding interactions described above and the fingerprint plot (Fig. 4d). The closest contacts are seen between hydrogen atoms and sulfur atoms (Fig. S9), and the fingerprint plot of AEPzH_2_^2+^ reveals strong –S $\cdots$ H– hydrogen bonding interactions as indicated by the needle-like features (Fig. 4d). While most close contacts are of the type –S $\cdots$ H–, the –H $\cdots$ H– interactions between two AEPzH_2_^2+^ ions are also significant, whereas only approx. 2 % of the observed interactions are –Sn $\cdots$ H-.

### Crystal structure of AEPz:EtOH-SnS-1

By mixing AEPz and ethanol in a 1:1 ratio by volume, a different compound of the R-SnS-1 family, i.e. AEPz:EtOH-SnS-1, was obtained. Using a solvent mixture is expected to modify the structure directing properties of the amine, e.g. through hydrogen bond formation. The crystalline product contains rhombic crystals (Fig. 2c) suitable for single crystal diffraction. The crystal quality is only moderate, but substantially higher than for crystals of AEPz-SnS-1. The structure of AEPz:EtOH-SnS-1 was solved in triclinic *P* $\overline{1 }$ (no. 2) (Table 1)*.*

**Figure 5:**
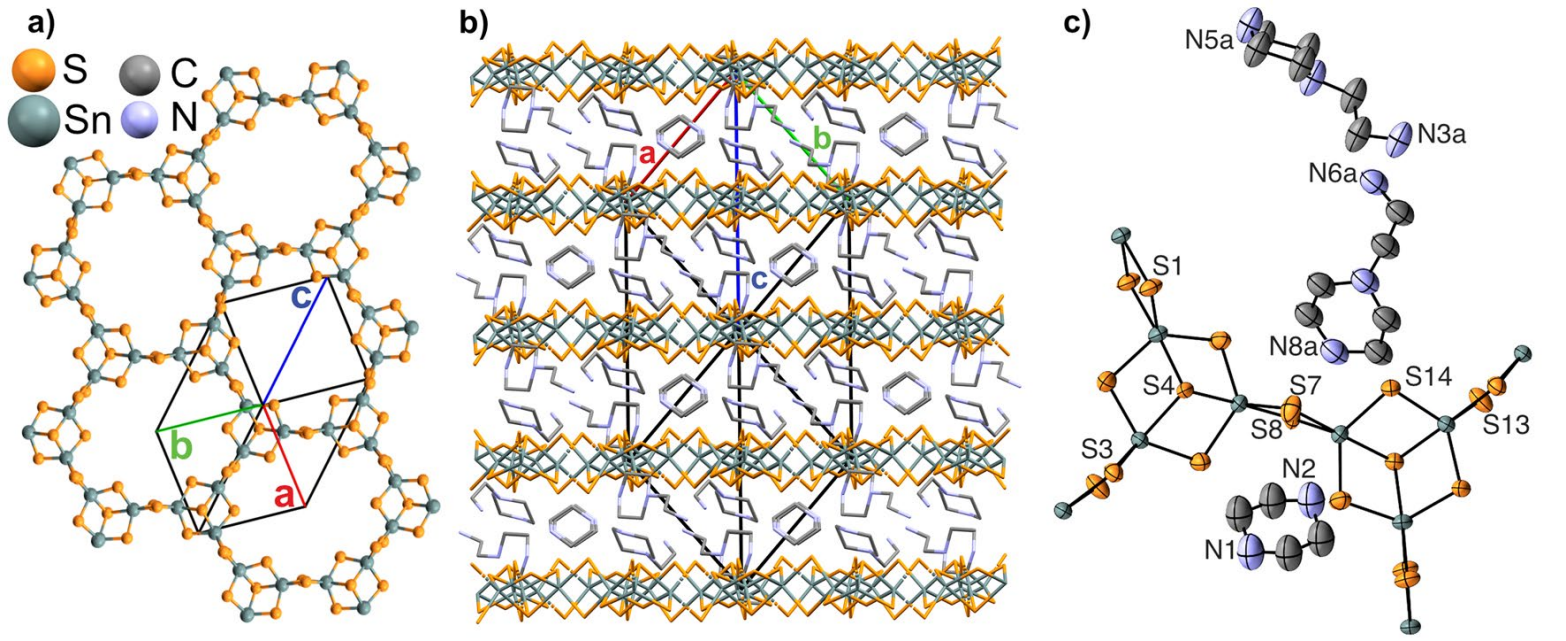
(**a**) Top view of a single [Sn_3_S_7_^2−^]_n_ layer showing non-regular hexagonal pores. (**b**) Side view of AEPz-SnS-1 showing the ordered molecular species (part *a*). (**c**) ORTEP representation (50% probability ellipsoids) with selected labels referring to close contacts in Table S6. In (**b**,**c**) the molecular disorder is not displayed for clarity. See Fig. S12 and S13 for details on the molecular disorder and for full atomic labelling.

AEPz:EtOH-SnS-1 also consists of layers with the stoichiometry [Sn_3_S_7_^2−^]_n_, but in contrast to AEPz-SnS-1, the pores are not regular hexagons (Fig. 5a), and the layers stack perpendicular to the (112) planes in an ABCDABCD sequence. Moreover, the organic molecular species in AEPz:EtOH-SnS-1 are crystallographically ordered in-between the [Sn_3_S_7_^2−^]_n_ layers (Fig. 5b). The structure contains two types of molecular organic species, namely AEPz and piperazine (Pz). The latter has formed in situ through cleavage of the AEPz aminoethyl group. The overall stoichiometry of the compound is Sn_3_S_7_·(AEPz)·0.5(Pz), but the positions of the hydrogen atoms, which is required to obtain charge neutrality of the compound, could not be identified from the electron density map.

The distance across one hexagonal pore between opposite bridging sulfur atoms vary between 11.739(9) and 14.56(1) Å, as compared to 13.13(4) Å in AEPz-SnS-1 with regular hexagonal pores. As a consequence of stronger cation–anion interactions, the inter-layer distance has decreased from 9.425(6) Å in AEPz-SnS-1 to 7.613(4) Å in AEPz:EtOH-SnS-1. This difference is reflected in the higher calculated density for AEPz:EtOH-SnS-1 of 2.21 g⋅cm^−1^ in comparison with 1.98 g⋅cm^−1^ for AEPz-SnS-1. The inter-layer distance in AEPz:EtOH-SnS-1 is also significantly shorter than the inter-layer spacing in the structurally similar compound AEP-SnS-1, which was reported to be 9.0772(2) Å^23^. Instead, it is similar to the inter-layer spacing in the compound FJSM-SnS-1 which is 7.403(2) Å at 100 K^32^. Interestingly, FJSM-SnS-1 contains the much smaller tri- and dimethylamine, indicating that strong cation–anion interactions are present in AEPZ:EtOH-SnS-1 despite of the large AEPz and Pz molecular species.

In AEPz:EtOH-SnS-1 the Sn-S bond lengths vary between 2.362(7) Å and 2.612(6) Å, while the S-Sn-S’ angles are 87.8(2)°–178.9(2)° in agreement with the trigonal pyramidal coordination of tin (Table S7). This is in good agreement with the coordination geometry of previously reported R-SnS-1 structures^21,23,34^. The position of AEPzH^+^ was refined as two parts (denoted *a* and *b*) with occupancies of 0.51 and 0.49, respectively, to describe positional disorder. Due to the moderate crystal quality (as mentioned above and described in detail in the ESI), the positions of the hydrogen atoms could not be determined from the diffraction data. All C and N atoms were restrained using EADP, DFIX and SAME commands in SHELX. A full list of bond distances and angles can be found in Table S7.

In the structure of AEPz:EtOH-SnS-1, the ammonium functionalities of the AEPzH^+^ cations interact strongly with the [Sn_3_S_7_^2−^]_n_ layers (Table S6). The strongest interactions are observed for the secondary amines (N5a/b and N8a/b) with –N $\cdots$ S– distances ranging from 3.16(3) to 3.36(3) Å (Fig. 5c). The corresponding –N $\cdots$ S– distances of the primary amino functionalities (N3a/b and N6a/b) are 3.44(5)-3.67(6) Å. Similarly, the secondary amino groups in the free-standing Pz ring (N1 and N2) also have strong interactions with the thiostannate layers with –N $\cdots$ S– distances of 3.40(3) and 3.90(3) Å. Although we cannot determine from direct structure solution which amino functionalities are protonated, through simple inspection of the –N $\cdots$ S– distances, we suggest that the following groups are protonated: (*i*) the secondary amino groups of AEPz (N5a/b and N8a/b), (*ii*) N2 on Pz, and (*iii*) one of the primary amino groups (N3a/b or N6a/b). This gives four ammonium groups per [Sn_3_S_7_^2−^]_2_, hence a net charge of zero.

Interestingly, despite of adding ethanol as a co-solvent, the alcohol was not incorporated into the structure according to CHNS analysis (see Methods section). It may, however, play a role on the cleavage of AEPz to Pz and/or have different structure directing properties than AEPz alone, e.g. through formation of hydrogen bonds. Understanding the deeper role of ethanol on the crystal formation mechanism was not investigated further herein and would require following the reaction in situ.

### Light absorption properties of the thiostannates

**Figure 6:**
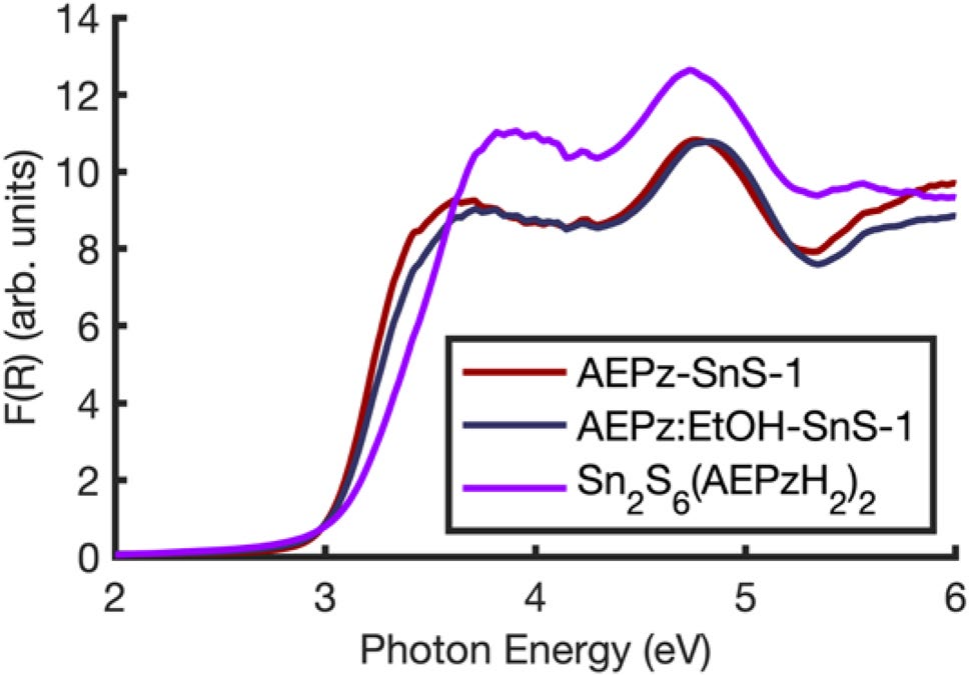
Kubelka–Munk transformed DRS data of AEPz-SnS-1, AEPz:EtOH-SnS-1, and Sn_2_S_6_(AEPzH_2_)_2_ as a function of photon energy. The spectra of AEPz-SnS-1 and AEPz:EtOH-SnS-1 are nearly identical, whereas the spectrum of Sn_2_S_6_(AEPzH_2_)_2_ is similar in shape, but shifted to slightly higher energies.

The band gaps of the three pristine materials were determined by diffuse reflectance spectroscopy (DRS). Kubelka–Munk transformed spectra are shown in Fig. 6 (and raw data are shown in Fig. S15) revealing large similarity in the light absorption properties of the three compounds. In agreement with our previous findings^33^, the band gap of AEPz-SnS-1 was determined to 3.0 eV (413 nm, Fig. S16a). This corresponds to violet light absorption and is comparable to the band gap of isostructural trenH-SnS-1^13^, but is somewhat lower than the band gap of 3.2 eV, which was observed for the thiostannate AEP-SnS-1 containing a similar amine, namely AEP = 1-(2-aminoethyl)piperidine^23^. Interestingly, despite the structural differences, AEPz:EtOH-SnS-1 also has a band gap of 3.0 eV (Fig. S16b), but the DRS data reveals larger absorption in the visible range, as the “edge” in the reflectance data is less steep for AEPz:EtOH-SnS-1 than for AEPz-SnS-1. Finally, the band gap of Sn_2_S_6_(AEPzH_2_)_2_ was determined to be 3.1 eV (400 nm, Fig. S16c), hence slightly larger than the R-SnS-1 bandgaps, but it is significantly lower than that of other dimeric thiostannates containing metal-free organic cations^27,28^. The material, however, absorbs some light in the visible range. The identical band gaps suggest that the ordering of the cations, the different structure of the [Sn_3_S_7_^2−^]_n_ layers, and the inter-layer spacing only have a limited effect on the optical properties of the layered compounds. The fact that the dimeric compound shows similar light absorption properties suggest that there is a complex interplay between structure and bandgap.

### Stability dependence of cation order in R-SnS-1

In a previous study, we discovered that two R-SnS-1 materials with disordered cations (AEPz-SnS-1 and trenH-SnS-1) undergo a phase transition from crystalline to amorphous when dispersed in water. Based on these observations we suggested that the cation disorder in R-SnS-1 compounds might play a role on their stability in water^33^. AEPz:EtOH-SnS-1 with ordered cations does not lose its crystallinity in water suspension according to PXRD (Fig. S17). This observation supports the hypothesis that cation order, which is indicative of strong dipolar interactions in the thiostannates, increases water stability.

**Figure 7:**
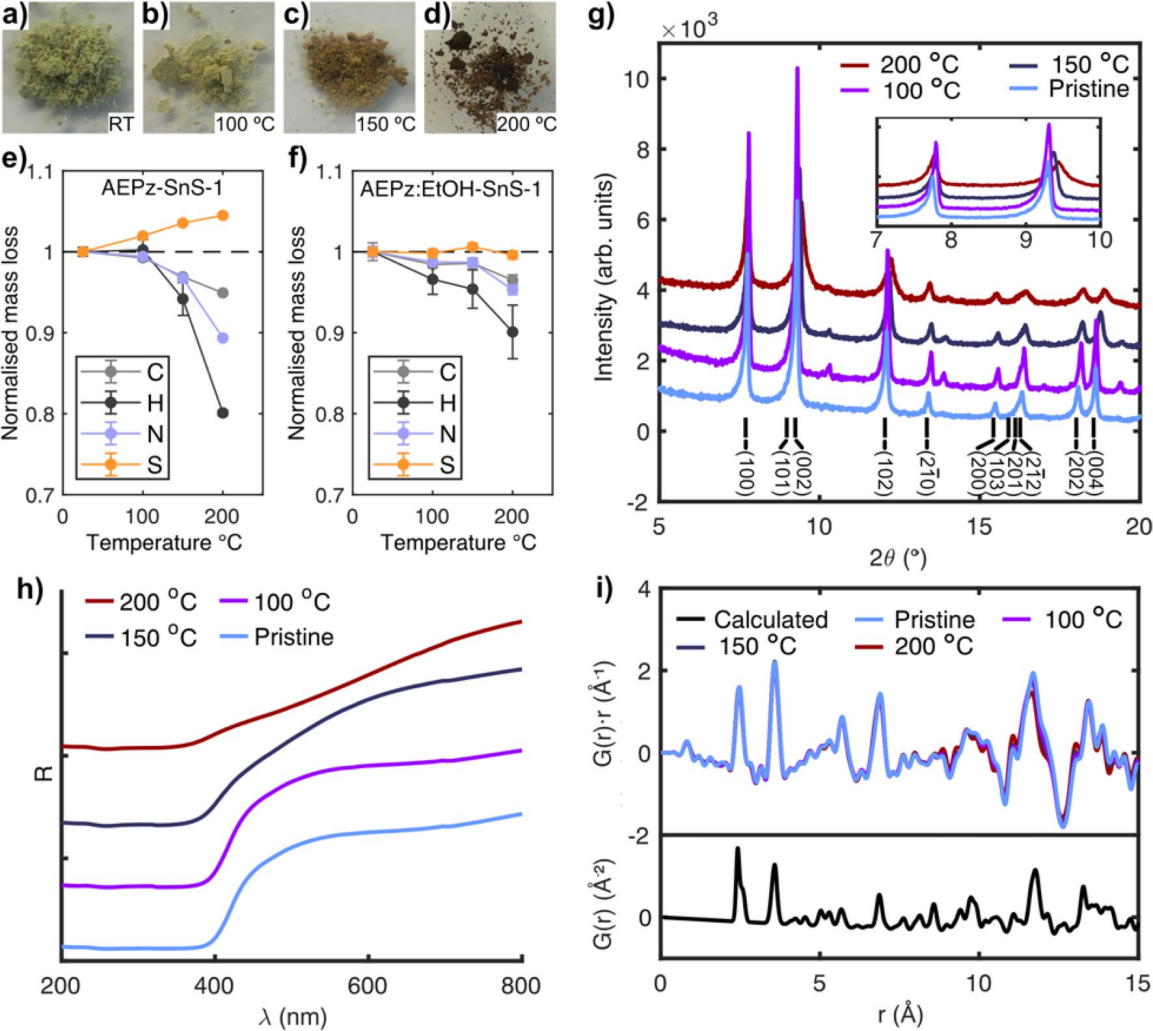
(**a**–**d**) AEPz-SnS-1 powder sample dried at room temperature (RT) and samples heated in air to target temperatures of 100 °C, 150 °C and 200 °C respectively. (**e**–**f**) CHNS analysis of AEPz-SnS-1 and AEPz:EtOH-SnS-1. Data are shown as weight fractions normalised to the respective pristine material. (**g**) PXRD data of all AEPz-SnS-1 samples with Miller indices based on CIF data. The insert is a zoom-in on the (100) and (101)/(002) peaks to highlight shifts in the diffraction angle and peak broadening of the (101)/(002) peak. (**h**) DRS data of the samples shown in (**a**–**d**). The spectra are shifted along the y-axis for easier comparison. No changes were observed at 100 °C. (**i**) PDFs of the heated samples of AEPz-SnS-1. *G*(*r*) is weighted by the correlation length (*r*) to highlight correlations at large distances in the experimental data. The calculated PDF of the pristine material is displayed as *G*(*r*).

The fact that the same structure directing amine uniquely resulted in the synthesis of R-SnS-1 type materials with cation disorder (AEPz-SnS-1) and order (AEPz:EtOH-SnS-1), respectively, motivated us to investigate the influence of cation order/disorder on the thermal stability and light absorption properties of the two layered compounds. The two R-SnS-1 compounds were heated to 100, 150 and 200 °C in air, after which they were cooled to room temperature and analysed. Interestingly, after heating to 100 °C, both compounds show a slight colour change, before they started to turn red-brown at 150 °C and dark red-brown at 200 °C (see AEPz-SnS-1 in Fig. 7a–d). As the materials are synthesised at 190 °C under solvothermal conditions, structural changes at these temperatures were unexpected. However, the differences are most likely due to the fact that heating in air offers oxidising conditions.

After heating to 200 °C, mass losses of approx. 8% and 3%, respectively, were observed for AEPz-SnS-1 and AEPz:EtOH-SnS-1. CHNS analysis confirms that AEPz-SnS-1 loses a significant amount of C, N and H, while the relative weight of S increases (Fig. 7e, Table S9). These results reveal that the mass loss associated with the inorganic thiostannate framework is minor compared to that of the organic substance. The same trend is also observed in AEPz:EtOH-SnS-1, albeit to a much smaller extent; the C, N and H contents decrease, while S retains a constant weight fraction (Fig. 7f, Table S9).

PXRD analysis at room temperature confirms that the structure of AEPz:EtOH-SnS-1 largely remains unchanged upon heating (Fig. S18). In contrast, PXRD data on AEPz-SnS-1 shows changes in the Bragg peak positions and peak broadening (Fig. 7g, see full diffractograms in Fig. S19). The Bragg peaks associated with the hexagonal AEPz-SnS-1 structure remain for all samples, despite the colour and mass change. However, the peaks are clearly broadened as an indication of increased strain in the materials, especially for the sample heated to 200 °C. For the samples prepared at 100 °C and 150 °C, additional low intensity Bragg peaks are observed (e.g. at 10° and 13°). These additional peaks might be indicative of a symmetry decrease caused by structural rearrangement, possibly of the cations. A number of unit cell transformations, e.g. from hexagonal to orthorhombic, were tested without success to describe the observed pattern of all Bragg peaks. Reflections with Miller indices containing *l* components gradually shift towards higher angles indicative of decreased inter-layer distances (Fig. 7g). Specifically, the (002) reflection (insert in Fig. 7g) shifts from 9.30° to 9.44° corresponding to inter-layer distances of 9.554(2) Å (pristine) and 9.355(8) Å (after heating to 200 °C) according to refinement of the PXRD data (Fig. S2 and S20, Table S2 and S10). The decreased inter-layer distance is in good agreement with the loss of organic matter. However, despite of the decrease, the inter-layer distance of 9.355(8) Å is still much larger than the AEPz:EtOH-SnS-1 inter-layer spacing of 7.613(4) Å.

As AEPz-SnS-1 shows structural changes upon heating, the light absorption properties were investigated further. DRS data of heated AEPz-SnS-1 are displayed in Fig. 7h (raw data are shown in Fig. S23). The pristine and the sample heated to 100 °C have identical spectra in the wavelength range 200–800 nm, with a sharp edge around 400 nm. For comparison, the sample prepared at 150 °C starts to show a broader absorbance edge stretching up to approx. 650 nm. Nonetheless, the lower edge remained at 400 nm, as for the pristine sample. The same behaviour, albeit more significant, is observed for the 200 °C sample. Thereby, the overall light absorption properties are similar to those of the pristine sample, but the absorption edge is much less pronounced after heating.

We speculate if the heat treatment, which involved a loss of the organic matter, has caused the samples to become partly amorphous. This might explain the observed changes in the light absorption properties, despite retaining the overall crystal structure according to PXRD. This motivated an investigation of the heat treated samples of AEPz-SnS-1 by pair distribution function (PDF) analysis based on X-ray total scattering data. Figure 7i shows PDFs of AEPz-SnS-1 represented as *G*(*r*)⋅*r*, which highlights subtle features at high *r* (see Fig. S21 for plots of *G*(*r*) and Fig. S22 for plots of *G*(*r*)⋅*r* in an extended range of *r*). Inspection of the PDF data below 8.5 Å confirms the in-layer local structure to be maintained for all samples, as no new peaks appear or disappear, and no significant shifts in peak positions are observed. Peaks at distances larger than the inter-layer distance show minor changes including some slightly decreasing correlation lengths, which is in agreement with the inter-layer distance contraction according to PXRD (Fig. 7g). For instance, the peak at 11.71 Å (correlation across two layers) in the pristine materials has shifted to 11.66 Å for the sample heated to 200 °C (Fig. 7i). However, as inter-layer as well as in-layer correlations contribute to the PDF at *r* > 9 Å, the PDFs at large distances are highly complex. Overall, however, no significant changes in the local thiostannate structure is observed.

To summarise, we propose that cation order has a significant impact on the material stability. This is observed by the increased water and heat stability of the ordered AEPz:EtOH-SnS-1. For comparison, suspension of AEPz-SnS-1 in water led to amorphization^33^, while heat treatment led to a substantial loss of organic matter. While the compounds were seen to either remain crystalline (AEPz:EtOH-SnS-1) or have an unchanged in-layer local structure (AEPz-SnS-1), CHNS reveals that the incorporated AEPz molecules are more easily evaporated from the disordered AEPz-SnS-1 relative to ordered AEPz:EtOH-SnS-1. We suggest evaporation of AEPz to be the main mechanism behind the decrease in inter-layer distance observed for AEPz-SnS-1, and the colour change may be due to surface oxidation or charring of residual organics on the surface from the evaporation process.

Overall, for the R-SnS-1 compounds we suggest that the light absorption of the pristine materials appears to be largely independent of cation order and symmetry of the hexagonal pores. However, cation order, which is indicative of strong guest molecule-thiostannate framework interactions, indeed affects the stability of the materials.

## Conclusion

In this study, we present synthesis protocols for three thiostannates by variation of temperature, stoichiometry and solvent. All procedures use AEPz as the structure directing agent and solvent. We have solved the crystal structures of two layered R-SnS-1 compounds (AEPz-SnS-1 and AEPz:EtOH-SnS-1) and molecular Sn_2_S_6_(AEPzH_2_)_2_. The compound Sn_2_S_6_(AEPzH_2_)_2_ was prepared at 150 °C and contains AEPzH_2_^2+^ and Sn_2_S_6_^4−^ motifs. AEPz-SnS-1 was obtained when using AEPz as the only solvent at 190 °C. In this compound, protonated cations of AEPz are intercalated between [Sn_3_S_7_^2−^]_n_ layers with regular hexagonal pores, and the cations are crystallographically disordered. When mixing AEPz with ethanol, AEPz:EtOH-SnS-1 was obtained at 190 °C with ordered cations between layers of [Sn_3_S_7_^2−^]_n_ with distorted hexagonal pores. Actually, in AEPz:EtOH-SnS-1 two different organic species are intercalated, namely protonated AEPz and piperazine formed in situ by cleavage of the aminoethyl group from AEPz. The ordering of the organic species reduced the inter-layer spacing by approx. 20% resulting in an increased density in comparison with AEPz-SnS-1. The three materials have band gaps in the range of 3.0–3.1 eV.

For the R-SnS-1 compounds, the influence of cation order on the thermal and water stability was investigated. The stronger cation–anion interactions in AEPz:EtOH-SnS-1 causes the material to be substantially more stable in air up to 200 °C and in water at room temperature in comparison with AEPz-SnS-1. Upon heating, the inter-layer distance of AEPz-SnS-1 was found to decrease by approx. 0.2 Å, and strain was induced in the crystalline powder, but the local structure was preserved. The decrease in inter-layer distance is associated with loss of AEPz, as observed by elemental analysis. We also conclude that the optical properties of the pristine materials have low dependence of the inter-layer distance and cation order, but the latter substantially modifies the material stability.

## Methods

### Synthesis

#### Chemicals

Elemental sulfur (≥ 99.9%), SnO_2_ (≥ 99.5%), 1-(2-aminoethyl)piperazine (C_6_H_15_N_3_, 99%, AEPz) and absolute ethanol (C_2_H_5_OH) were purchased from Sigma-Aldrich and used without further purification.

#### Solvothermal synthesis of pristine thiostannates

AEPz-SnS-1, Sn_2_S_6_(AEPzH_2_)_2_ and AEPz:EtOH-SnS-1 were synthesised similarly to our previously reported description using stainless steel autoclaves with a 23 mL Teflon liner^33,36^. For all syntheses, 2 mL AEPz and 0.2 g sulfur were used, while the amount of SnO_2_ was adjusted according to the stoichiometry of the desired products. The precursor mixtures were stirred in the Teflon liner for ~ 5 min, before the autoclave was sealed and placed in a convection oven (Memmert UF-30plus) for 6 days at the target temperature. Subsequently the oven was turned off, and the autoclaves were cooled to room temperature over approx. 4 h, unless otherwise specified. Finally, the powders were isolated by suction filtration, washed with absolute ethanol, and dried in air.

AEPz-SnS-1 was synthesised by mixing SnO_2_ (0.35 g, 2.3 mmol) and sulfur (0.2 g, 6.2 mmol), i.e., in a molar ratio of 3:8, at 190 °C. Approx. 0.6 g was obtained (yield 90% based on Sn). To improve the growth of single crystals for structure determination, a 30-hour linear cooling ramp from 190 °C to room temperature was applied, following the 6 days at 190 °C﻿.

Compound Sn_2_S_6_(AEPzH_2_)_2_ was synthesised at 150 °C using SnO_2_ (0.1 g, 0.66 mmol) and sulfur (0.2 g, 6.2 mmol) corresponding to a molar ratio of ~ 1:10. Approx. 0.15 g was obtained (yield 64% based on Sn).﻿

 To prepare AEPz:EtOH-SnS-1, SnO_2_ (0.40 g, 2.6 mmol) and sulfur (0.2 g, 6.2 mmol) were heated to 190 °C in a molar ratio of 3:7, and a solvent mixture of 2 mL AEPz and 2 mL absolute ethanol was used. 0.6 g product was obtained (90% based on Sn). CHNS found/calculated % for AEPz:EtOH-SnS-1: C, 12.5(1)/12.7%; H, 3.04(1)/2.94%; N, 7.10(5)/7.42%; S, 25.2(1)/29.7%. Calculated values are based on the sample stoichiometry according to a composition of Sn_6_S_14_·C_16_N_8_H_42_ (Table 1).

#### Heat-treated AEPz-SnS-1

Using the as-synthesised AEPz-SnS-1 and AEPz:EtOH-SnS-1 samples, approx. 0.2 g sample was placed in a convection oven at room temperature. A linear heating ramp was used to reach the target temperature (100, 150 or 200 °C) in 1 h. Following, the sample was heated at the target temperature for 1 h, and the oven was subsequently ramped down to room temperature over 1 h.

### Powder X-ray diffraction

A Rigaku SmartLab diffractometer using Cu *K*α_1_ radiation (*λ* = 1.54056 Å) and a D/TEX Ultra 250 multi-channel detector was used for collection of PXRD data. Samples were packed in glass capillaries (inner diameter 0.5 mm) and convergence beam geometry was applied. The FullProf suite was used for Le Bail and Rietveld refinements^40^.

### Single crystal X-ray diffraction

Single crystal X-ray diffraction data on Sn_2_S_6_(AEPzH_2_)_2_ were acquired on a Bruker Apex-II CCD diffractometer using monochromatic Ag *K*α radiation (*λ* = 0.56086 Å). The Apex-III software was used for data integration and correction, specifically data were corrected for absorption in SADABS-2016/2 by applying a multi-scan method^41^. Data on crystals of AEPz-SnS-1 and AEPz:EtOH-SnS-1 were measured on an Oxford Diffraction Supernova diffractometer with a molybdenum microfocus sealed X-ray tube (Mo *K*α radiation, *λ* = 0.71073 Å), and an Atlas CCD detector. The CrysAlisPro software (version 1.171.40.67a) was used for integration, and a spherical absorption correction was performed in ABSPACK (included in the CrysAlisPro software package). Crystals of Sn_2_S_6_(AEPzH_2_)_2_ were cooled to 100 K during data collection using a nitrogen cooler from Oxford Cryosystems, whereas data on AEPz-SnS-1 and AEPz:EtOH-SnS-1 were measured at room temperature due to cracking of the crystals upon cooling. The structures were solved using SHELXT^42^ or SHELXS and refined against *F*^2^ in SHELXL^43,44^ by using the Olex2 interface^45^. The SQUEEZE procedure was used in the analysis of AEPz-SnS-1 data to deal with disordered organic substances^39^.

Hirshfeld surface analysis was carried out using the CrystalExplorer software^46^. Hirshfeld surface analysis was used to analyse molecular interactions between AEPzH_2_^2+^ and Sn_2_S_6_^4−^ in Sn_2_S_6_(AEPzH_2_)_2_. Close contacts were visualised through a fingerprint plot, which represents molecular interactions in a two-dimensional map. Along the abscissa is plotted the distance from the Hirshfeld surface to the nearest internal atom (*d*_i_), while the distance from the surface to the nearest external atom (*d*_e_) is plotted along the ordinate. For molecules with hydrogen bonding, sharp needle-like features are expected^47^.

### X-ray total scattering and pair distribution function

X-ray Total scattering data were collected at beamline P02.1 at PETRAIII at the Deutsches Elektronen-Synchotron (DESY), Germany, using an energy of 60 keV (*λ* = 0.2072 Å, beam size 1 × 1 mm). All samples were packed in Kapton capillaries (inner diameter 1.0 mm) and placed in a home-built sample changer. Scattered intensities were collected on a 2D detector (Perkin Elmer XRD1621) at a sample-to-detector distance of ~ 20 cm (calibrated using LaB_6_). For each sample, data were measured for 5 × 1 min in 1 s exposures, and the 1 min frames were integrated and subsequently summed using Dioptas V0.4^48^. Thereby, data in the *Q*-range of 0.5–20 Å^−1^ were obtained. The total scattering data were background corrected by subtraction of the scattered intensities from an empty Kapton capillary. The corrected data were transformed to Pair Distribution Functions (PDF), *G*(*r*), using xPDFsuite^49^ based on the PDFGetX3 engine^50^. Finally, pattern calculations and refinements were performed using PDFgui^51^.

### Scanning electron microscopy

Scanning electron microscopy (SEM) images were captured using a FEI-Nova Nano SEM 600 in high vacuum. Samples were immobilized on double-sided carbon tape and coated with approx. 10 nm platinum to prevent charging. The platinum layers were deposited using a LEICA EM SCD 500 vacuum film deposit system equipped with a LEICA EM QSG100 Quartz Crystal Film Thickness Monitor to track the film thickness.

### Diffuse reflectance spectroscopy

Diffuse reflectance spectroscopy (DRS) data were measured on the samples in the range 200–800 nm in 1 nm steps on the Shimadzu UV-3600 spectrophotometer against BaSO_4_ as a reference. Band gaps of the materials were determined by transforming the reflectance data using the Kubelka–Munk function, where *R* is the reflectance, and *F* is the transformed data.

$$F=\frac{{\left(1-R\right)}^{2}}{2R}$$

### Elemental analysis

Elemental analyses of AEPz:EtOH-SnS-1 and AEPz-SnS-1 and their heat treated samples were performed on a Elementar Vario MACRO cube in CHNS mode. Sulfanilamide was used as a reference (approx. 25 mg for each measurement). Triplicates were analysed with approx. 20–25 mg sample and approx. 10 mg WO_3_ as catalyst.

## Supplementary Information

Supplementary Information﻿.

## Data Availability

CIF-files of AEPz-SnS-1, Sn2S6(AEPzH2)2, and AEPz:EtOH-SnS-1 have been deposited to the CCDC database (no. 2057280- 2057282).
