# Supplementary material for: Exploring the structures, stability, and light absorption properties of three thiostannates synthesised at similar conditions
Source: Sci Rep. 2021 Nov 11;11:22080. doi: 10.1038/s41598-021-01329-9 (PMC8586010; doi:10.1038/s41598-021-01329-9)
Supplement: Supplementary file 1 — Supplementary Information﻿. [file 41598_2021_1329_MOESM1_ESM.pdf]

Electronic Supplementary Information

Exploring the structures, stability and light absorption properties of three thiostannates synthesised at similar conditions

Henrik S. Jeppesen[1,2], Peter Nørby[3], Jens Jakob Gammelgaard[1], Kasper Borup[3], Nina Lock[4]*

[1]Interdisciplinary Nanoscience Center (iNANO), Aarhus University, Gustav Wieds Vej 14, DK-8000 Aarhus C, Denmark.

[2]Sino-Danish Center for Research and education (SDC).

[3]Center for Materials Crystallography (CMC), Department of Chemistry, Aarhus University, Langelandsgade 140, DK-8000 Aarhus C, Denmark.

[4]Carbon Dioxide Activation Center (CADIAC), Department of Biological and Chemical Engineering, Aarhus University, Åbogade 40, DK-8200 Aarhus N, Denmark

*corresponding author: nlock@bce.au.dk

Table of Contents

1. **Compound: AEPz-SnS-1** 3
   - 1.1 *Optimizing crystal growth and collection of single crystal diffraction data* 3
   - 1.2 *Low magnification SEM of AEPz-SnS-1* 4
   - 1.3 *Phase identification of AEPz-SnS-1* 5
   - 1.4 *AEPz-SnS-1 crystal structure* 6
   - 1.5 *Rietveld refinements of AEPz-SnS-1* 6
2. **Compound: $Sn_2S_6(AEPzH_2)_2$** 8
   - 2.1 *Low magnification SEM of $Sn_2S_6(AEPzH_2)_2$* 8
   - 2.2 *Phase identification of $Sn_2S_6(AEPzH_2)_2$* 9
   - 2.3 *Rietveld refinements of $Sn_2S_6(AEPzH_2)_2$* 10
   - 2.4 *$Sn_2S_6(AEPzH_2)_2$ synthesis optimization* 12
   - 2.5 *$Sn_2S_6(AEPzH_2)_2$ crystal structure* 13
   - 2.6 *Hirshfeld surface of $AEPzH_2^{2+}$ molecular cations in $Sn_2S_6(AEPzH_2)_2$* 15
3. **Compound: AEPz:EtOH-SnS-1** 16
   - 3.1 *Optimizing crystal growth and collection of single crystal diffraction data* 16
   - 3.2 *Low magnification SEM of AEPz:EtOH-SnS-1* 16
   - 3.3 *Phase identification of AEPz:EtOH-SnS-1* 17
   - 3.4 *AEPz:EtOH-SnS-1 crystal structure* 18
   - 3.5 *Rietveld refinements of AEPz:EtOH-SnS-1* 24
4. **Diffuse reflectance spectroscopy of pristine compounds** 26
5. **Stability studies of AEPz-SnS-1 and AEPz:EtOH-SnS-1** 28
   - 5.1 *AEPz:EtOH-SnS-1 water treatment* 28
   - 5.2 *CHNS analysis of AEPz-SnS-1 and AEPz:EtOH-SnS-1* 29
   - 5.3 *AEPz:EtOH-SnS-1 thermal treatment* 29
   - 5.4 *Le Bail fits to PXRD data of heat treated AEPz-SnS-1* 30
   - 5.5 *Pair distribution function analysis of heat treated AEPz-SnS-1* 33
   - 5.6 *Diffuse reflectance spectroscopy of heat treated samples* 35
6. **References** 36

1. Compound: AEPz-SnS-1

1.1 Optimizing crystal growth and collection of single crystal diffraction data

Multiple attempts of increasing crystal quality and size was attempted by varying stoichiometry and temperature, by adding cooling ramps, and by addition of seed crystals. The synthesis conditions presented in the manuscript, which involved a cooling ramp, improved crystal quality. However, this procedure gave an impurity of $Sn_2S_6(AEPzH_2)_2$, as shown in Fig. S1.

Multiple single crystals were tested, all with similar outcomes in terms of unit cell. Upon cooling from room temperature to 100 K, crystal cracking was observed, and we decided to collect data for structure solution at room temperature. Despite of the moderate crystal quality and data, the structure of the thiostannate layers in AEPz-SnS-1 was solved based on single crystal diffraction data. A very high R_{int} (22.5%) was observed despite the low resolution of d_{min}=1.3 Å and average I/σ of 20.6 for all reflections up to 1.3 Å. We suspect that the low data quality is due to stacking faults and turbostratic disorder in the layers as observed by peak tailing in PXRD patterns and a rapid loss of long range order in pair distribution function analysis[1].

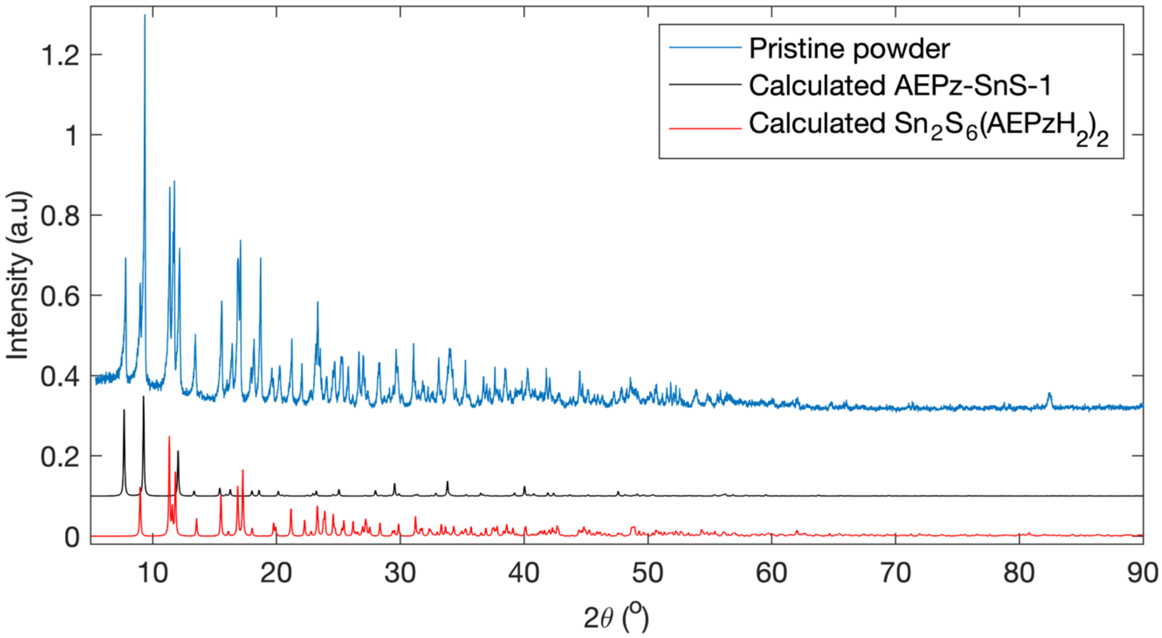

Fig. S1: PXRD of AEPz-SnS-1 synthesized under conditions aiming for increased crystal quality using a 30-hour linear cooling ramp. AEPz-SnS-1 with impurities of $Sn_2S_6(AEPzH_2)_2$ and SnO_2 resulted from this procedure.

1.2 Low magnification SEM of AEPz-SnS-1

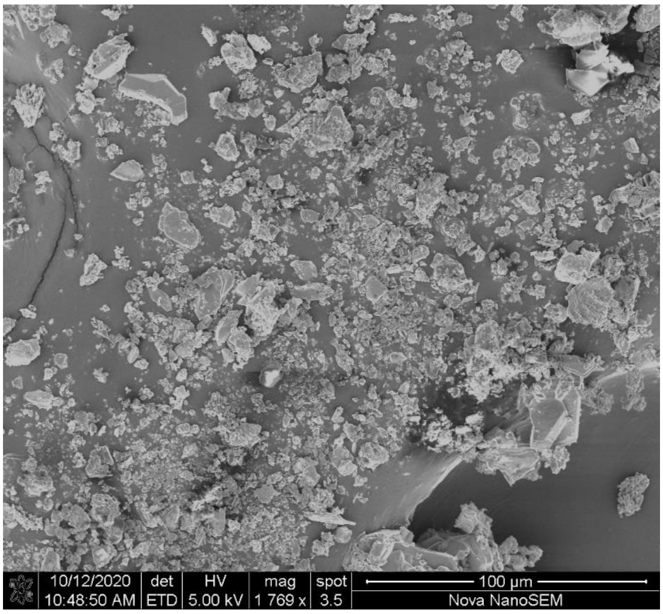

Fig. S2: Low magnification SEM of a sample of AEPz-SnS-1 synthesized with a 30-hour cooling ramp, i.e. an impurity of the dimer is present.

1.3 Phase identification of AEPz-SnS-1

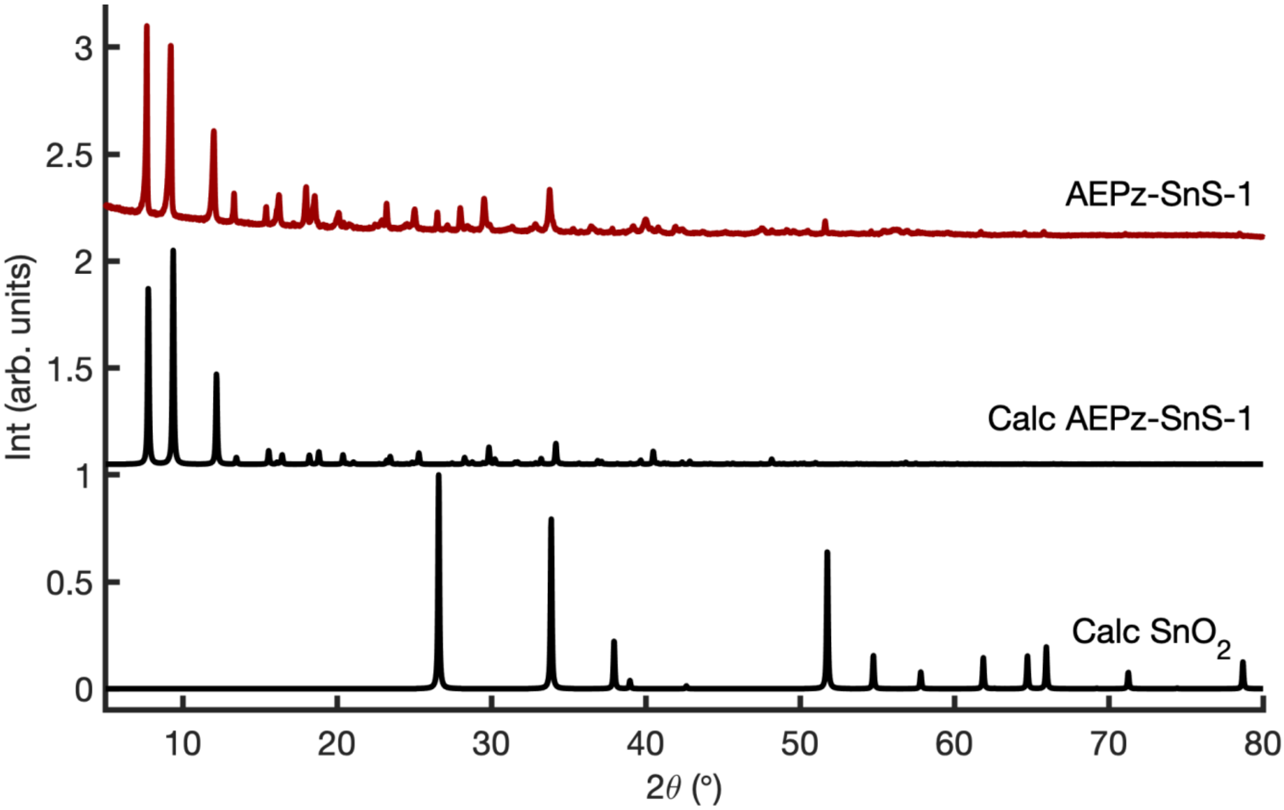

Fig. S3: Calculated patterns (black) of SnO_2 (ICSD entry 9163[2]) and AEPz-SnS-1 based on the CIF data obtained in this work. Experimental PXRD data of AEPz-SnS-1 (red) used for Rietveld refinement (as presented in the manuscript).

1.4 AEPz-SnS-1 crystal structure

The structure of AEPz-SnS-1 (isostructural to trenH-SnS-1[3,4]), is shown in the manuscript Fig. 1. Due to the disordered nature of the protonated AEPz molecules between the layers, only the bonding characteristics of the $[Sn_3S_7^{2-}]_n$ layers are shown in Table S1. The table was generated using PublCIF[5].

Table S1: Geometric parameters for AEPz-SnS-1 (Å, º).

**Sn1—S3**	2.603 (17)	**Sn1—S1^{ii}**	2.469 (14)
**Sn1—S2^{i}**	2.37 (2)	**Sn1—S1**	2.469 (15)
**Sn1—S2**	2.486 (19)		
**S2^{i}—Sn1—S3**	93.3 (7)	**S1—Sn1—S2**	90.4 (5)
**S2—Sn1—S3**	176.9 (7)	**S1^{ii}—Sn1—S1**	114.0 (9)
**S2^{i}—Sn1—S2**	89.8 (7)	**Sn1—S3—Sn1^{ii}**	87.4 (7)
**S2^{i}—Sn1—S1**	123.0 (5)	**Sn1—S3—Sn1^{iii}**	87.4 (7)
**S2^{i}—Sn1—S1^{ii}**	123.0 (5)	**Sn1^{iii}—S3—Sn1^{ii}**	87.4 (7)
**S1^{ii}—Sn1—S3**	87.9 (5)	**Sn1^{i}—S2—Sn1**	90.2 (7)
**S1—Sn1—S3**	87.9 (5)	**Sn1^{iii}—S1—Sn1**	93.5 (7)
**S1^{ii}—Sn1—S2**	90.4 (5)		

Symmetry codes: (i) −*x*+2, −*y*+1, −*z*+1; (ii) −*y*+1, *x*−*y*, *z*; (iii) −*x*+*y*+1, −*x*+1, *z*.

1.5 Rietveld refinements of AEPz-SnS-1

Rietveld refinements of PXRD data collected on AEPz-SnS-1 were performed in order to estimate the SnO_2 content in the sample.

CIF data for AEPz-SnS-1 and SnO_2 (ICSD entry 9163[2]) were used, and the atomic coordinates were fixed throughout the refinement. Absorption correction was applied in the refinement. The absorption was estimated to be μR = 2.76 based on the material density (1.3 g/cm^3 according to CIF) and a packing degree of 50% in the capillary. The background was described by manual selection of background points. The peak profile was described using a Thompson-Cox-Hastings pseudo-Voigt function with axial divergence asymmetry. Only *X* and *W* were refined whereas *U* and *V* were fixed to 0.00413 and -0.00762. Asymmetry was accounted for by refinement of the Asym1 parameter.

The refined pattern is shown in Fig. S4, and the refined parameters are summarised in Table S2. The fit is clearly underestimating the intensity of multiple peaks suggesting that the model from single crystal diffraction is insufficient to describe the powder diffraction data. Due to the large difference between data and model, the estimated SnO_2 content of 8% by mass should be seen as an approximate value only.

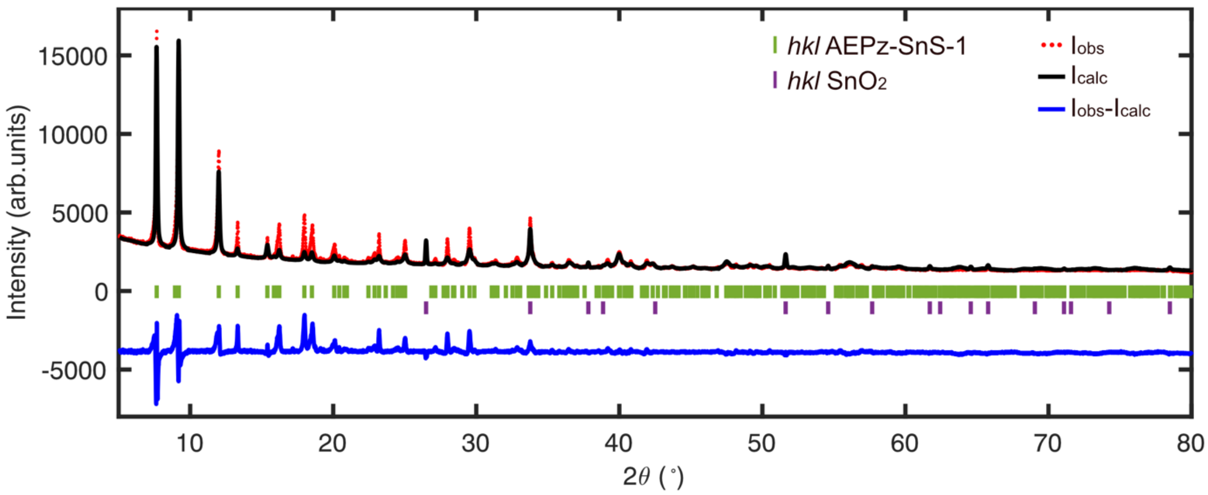

Fig. S4: Rietveld refinement of AEPz-SnS-1 and SnO_2. Measured intensities: red dots, calculated pattern: black line, difference curve (I_{obs}-I_{calc}): blue, Bragg positions of AEPz-SnS-1: green, and Bragg positions of SnO_2: purple.

Table S2: Refined parameters for AEPz-SnS-1 and SnO_2. Numbers marked by an asterisk (*) denote non-zero values that were not refined. (**) denote values that were refined and then fixed

	SnO_2	*AEPz-SnS-1*
Bragg R-factor (%)	21.2	49.9
R_F-factor (%)	11.9	28.3
Fraction (%)	8.2(6)	92(1)
	**Refined parameters**	
Zero	-0.041**	
Scale factor	$1.74(12)\cdot10^{-2}$	$6.29(4)\cdot10^{-4}$
a (Å)	4.7469(3)	13.2445(10)
c (Å)	3.1919(4)	19.1152(12)
U	0.00413*	0.00413*
V	-0.00762*	-0.00762*
W	0.008(2)	0.0067(4)
X	0.074(47)	1.02(3)
Asym1	-	0.015(2)
Overall *B*-factor (Å^2)	1.0*	1.0*

2. Compound: $Sn_2S_6(AEPzH_2)_2$

2.1 Low magnification SEM of $Sn_2S_6(AEPzH_2)_2$

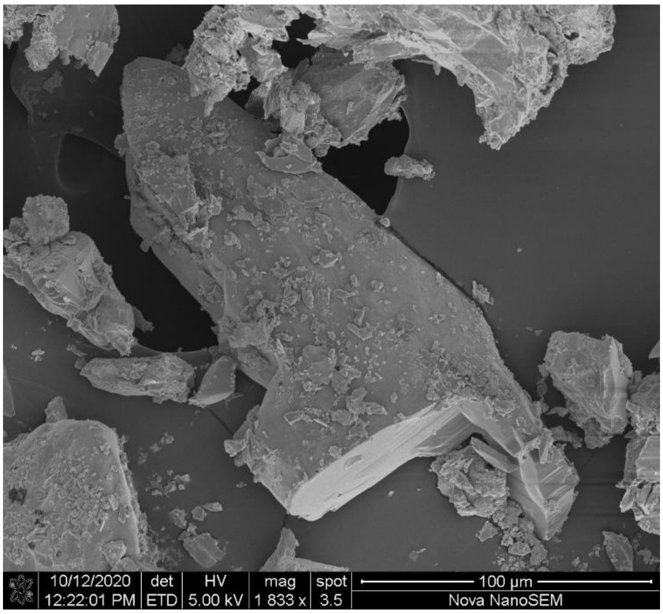

Fig. S5: Low magnification SEM of a sample of $Sn_2S_6(AEPzH_2)_2$.

2.2 Phase identification of $Sn_2S_6(AEPzH_2)_2$

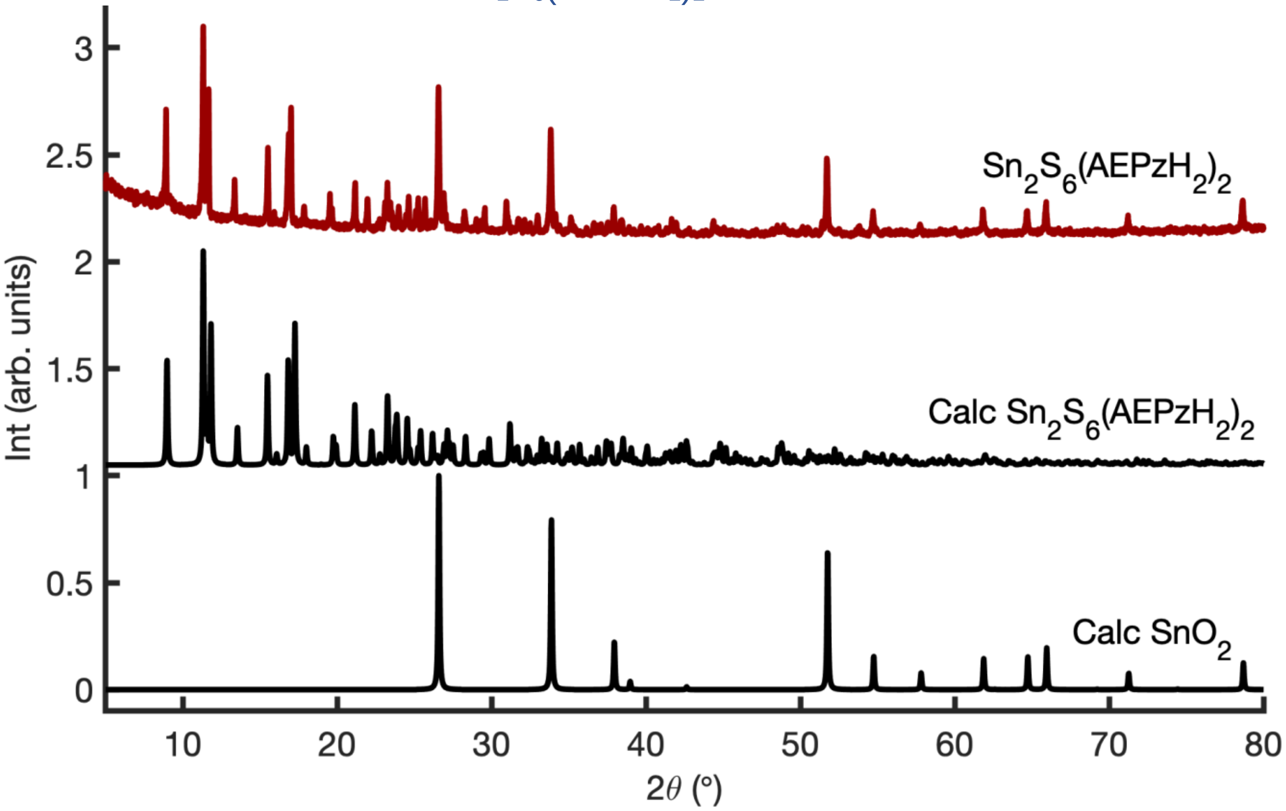

Fig. S6: Calculated patterns (black) of SnO_2 (ICSD entry 9163[2]) and $Sn_2S_6(AEPzH_2)_2$ based on the CIF data obtained in this work. Experimental PXRD data of $Sn_2S_6(AEPzH_2)_2$ (red) used for Rietveld refinement (as presented in the manuscript).

2.3 Rietveld refinements of $Sn_2S_6(AEPzH_2)_2$

Rietveld refinements of $Sn_2S_6(AEPzH_2)_2$ were performed in order to estimate the SnO_2 content in the sample.

CIF data for $Sn_2S_6(AEPzH_2)_2$ and SnO_2 (ICSD entry 9163[2]) were used as structural models. The absorption was estimated to be $\mu R = 2.40$ based on the material density (1.8 g/cm^3 according to CIF) and a packing density of 50%. The peak profiles were described using a Thompson-Cox-Hastings pseudo-Voigt function with axial divergence asymmetry, and only X and W were refined, whereas U and V were restrained to 0.00413 and -0.00762. Both phases were refined using an overall B-factor, and asymmetry was refined by Asym1 and Asym2. Finally, the position of oxygen (O1) was refined in SnO_2 and positions of tin (Sn1) and sulfur (S1-S3) were refined in $Sn_2S_6(AEPzH_2)_2$.

The resulting fit is shown in Fig. S7, and the refined parameters are summarized in Table S3. Based on the refinement, the SnO_2 impurity is estimated to be approximately 12% by mass.

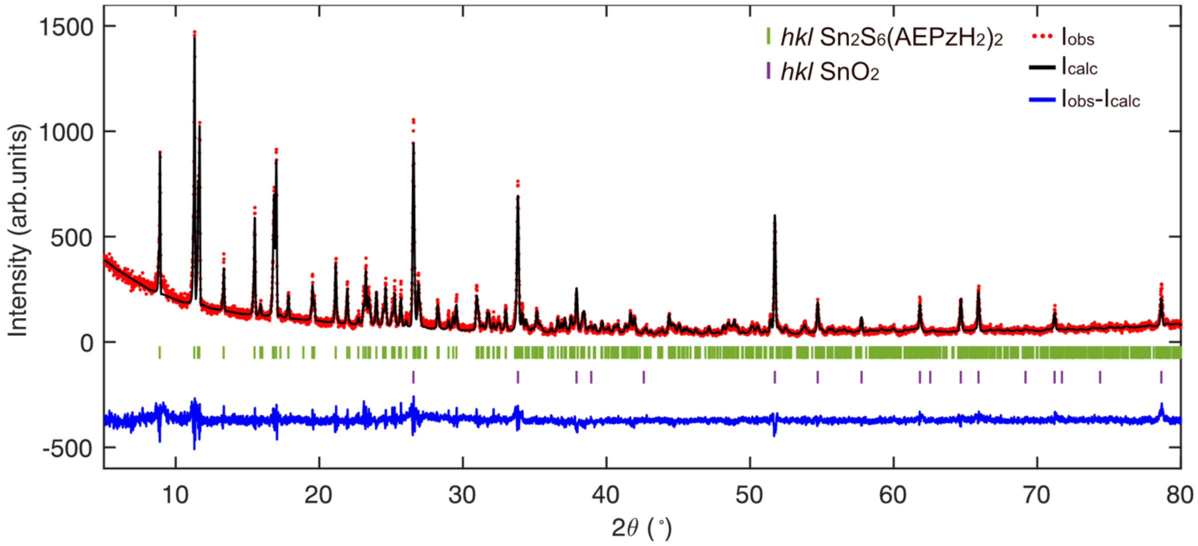

Fig. S7: A structural model for $Sn_2S_6(AEPzH_2)_2$ was obtained from structure solution based on single crystal diffraction (this paper), while CIF data for SnO_2 was obtained from ICSD entry 9163. The refinement is displayed as measured intensities (red dots), calculated pattern (black), difference curve I_{obs}-I_{calc} (blue), Bragg positions for $Sn_2S_6(AEPzH_2)_2$ (green) and SnO_2 (purple) are shown as vertical lines.

Table S3: Refined paramters for $Sn_2S_6(AEPzH_2)_2$ and SnO_2. Numbers marked by an asterisk (*) denote non-zero values that were not refined.

	SnO_2	*$Sn_2S_6(AEPzH_2)_2$*
Bragg R-factor (%)	9.95	16.2
R_F-factor (%)	6.62	13.0
Fraction (%)	11.5(1)	88.5(1.0)
	**Refined parameters**	
Zero	-0.0185(9)	
Scale factor	$6.797(58)\cdot10^{-3}$	$2.530(21)\cdot10^{-3}$
a (Å)	4.73999(6)	7.70992(18)
b (Å)	-	8.39728(19)
c (Å)	3.18857(5)	10.79275(27)
α (º)	-	110.8306(18)
β (º)	-	99.0390(21)
γ (º)	-	91.6350(14)
U	0.00413*	0.00413*
V	-0.00762*	-0.00762*
W	0.01893(22)	0.00972(17)
X	0.018*	0.2307(72)
Asym1	-	0.0876(33)
Asym2	-	0.0569(10)
Overall *B*-factor (Å^2)	0.766(68)	4.32(12)
	**Refined atomic positions**	
O1	*x* = 0.32023(154)	
Sn1	-	*x*=0.45745(61)<br>*y*=1.02196(69)<br>*z*=1.15985(42)
S1	-	*x*=0.1821(16)<br>*y*=0.9070(16)<br>*z*=1.1844(13)
S2	-	*x*=0.6685(16)<br>*y*=1.1533(16)<br>*z*=1.3629(14)
S3	-	*x*=0.4130(18)<br>*y*=1.2047(17)<br>*z*=1.0168(16)

2.4 $Sn_2S_6(AEPzH_2)_2$ synthesis optimization

Mixtures of $Sn_2S_6(AEPzH_2)_2$ and AEPz-SnS-1 may result by varying the stoichiometry of the SnO_2 and S precursors. Fig. S8 shows PXRD data of samples prepared from different precursor stoichiometries. For all syntheses, S (0.20 g, 6.2 mmol) and 2 mL of AEPz were heated to 150 °C with SnO_2 in the following Sn:S ratios: Sn:S = 3:7, SnO_2 (0.40 g, 2.6 mmol); Sn:S = 2:6, SnO_2 (0.32 g, 2.1 mmol), and Sn:S 1:7; SnO_2 (0.19 g, 1.3 mmol).

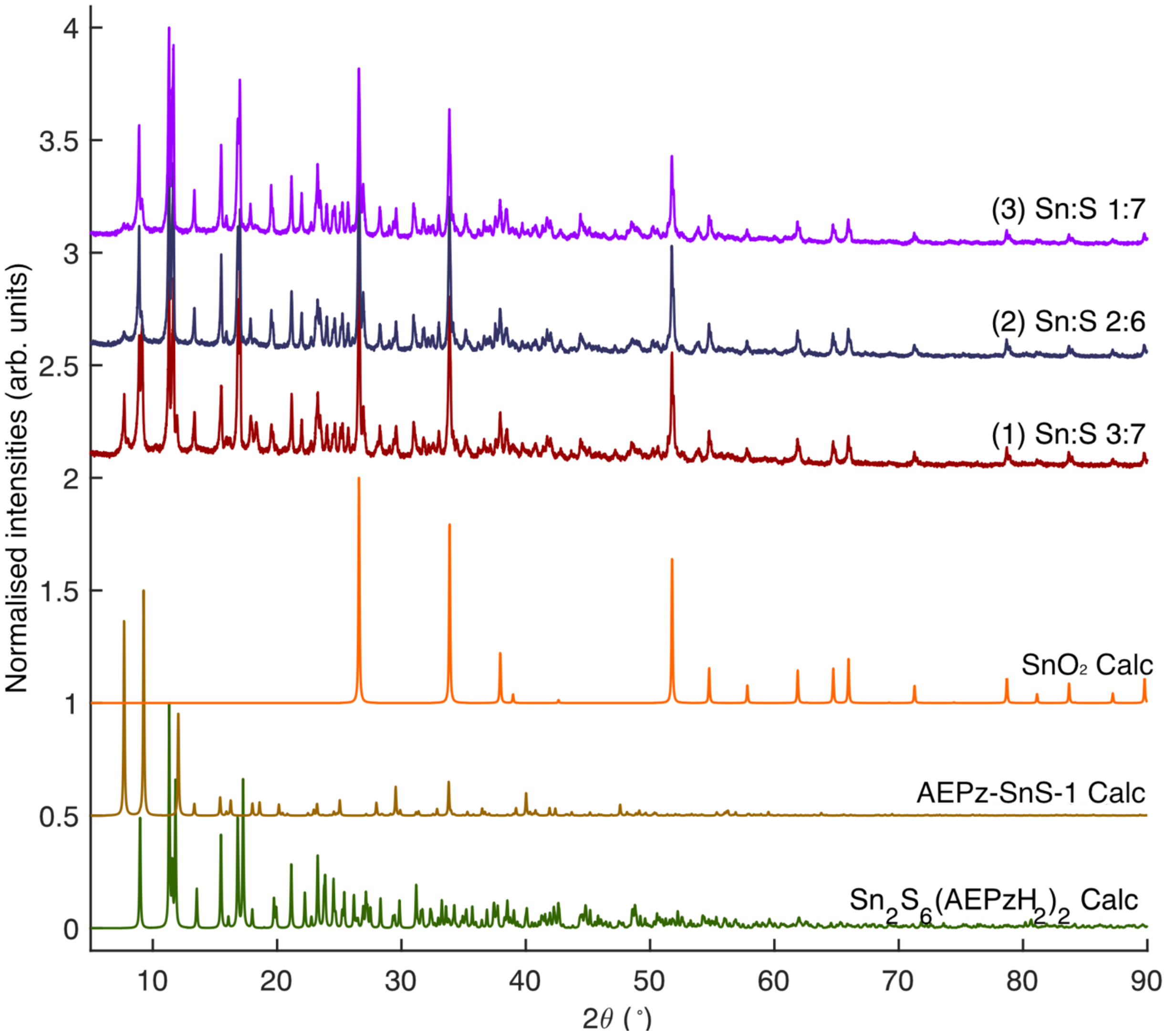

Fig. S8: PXRD data of products obtained by variation of the SnO_2:S precursor stoichiometry. All syntheses were performed at 150 °C. PXRD data were measured on a Malvern-Panalytical Aeris diffractometer (Cu $K\alpha$ radiation) using a flat sample holder in Bragg-Brentano geometry, and the most intense peak of each diffractogram was normalised to 1 and shifted along the ordinate. The three top patterns are experimental diffractograms of samples prepared at Sn:S ratios of 3:7 (dark red), 2:6 (navy blue) and 1:7 (purple).The three bottom patterns are calculated based on CIF data for $Sn_2S_6(AEPzH_2)_2$ (green), AEPz-SnS-1 (dark yellow), and SnO_2 (orange, ICSD entry 9163[2]).

2.5 $Sn_2S_6(AEPzH_2)_2$ crystal structure

Table S4 and Table S5 summarizes the hydrogen bonding and selected bond lengths, angles and torsion angles for $Sn_2S_6(AEPzH_2)_2$ as generated in PublCIF[5]. Data are based on the refinement of SCXRD data collected at 100 K using Ag Kα radiation.

Table S4: Hydrogen bonding distances and angles of $Sn_2S_6(AEPzH_2)_2$. Labels refer to Fig. 4b, and N1 is the primary amine, whereas N3 is the secondary amine.

	**-S···H-**	**-S···H-N-**
**-N3-H3C···S2'-**	2.29(3) Å	164(3)°
**-N3-H3D···S2-**	2.51(3) Å	161(2)°
**-N1-H1C···S1-**	2.45(3) Å	159(2)°
**-N1-H1D···S1'-**	2.41(3) Å	175(2)°
**-N1-H1E···S2'-**	2.64(3) Å	155(2)°

Table S5: Selected geometric parameters (Å, º) of $Sn_2S_6(AEPzH_2)_2$.

**Sn1—S1**	2.3291 (3)	**C4—C5**	1.508 (2)
**Sn1—S2**	2.3412 (3)	**C4—H4A**	0.96 (3)
**Sn1—S3**	2.4297 (3)	**C4—H4B**	0.94 (2)
**Sn1—S3**^{i}	2.4454 (3)	**C5—H5A**	0.95 (2)
**N1—H1A**	0.88 (3)	**C5—H5B**	1.00 (3)
**N1—H1B**	0.86 (2)	**C6—H6A**	1.01 (3)
**N1—C4**	1.4849 (17)	**C6—C7**	1.504 (2)
**N1—H1C**	0.86 (2)	**C6—H6B**	0.99 (3)
**N2—C5**	1.4599 (17)	**C8—H8A**	1.01 (2)
**N2—C6**	1.4597 (18)	**C8—C9**	1.508 (2)
**N2—C8**	1.4625 (18)	**C8—H8B**	1.03 (3)
**N3—H3A**	0.99 (3)	**C7—H7A**	0.99 (3)
**N3—H3B**	0.87 (2)	**C7—H7B**	0.96 (3)
**N3—C7**	1.485 (2)	**C9—H9A**	1.00 (3)
**N3—C9**	1.492 (2)	**C9—H9B**	1.01 (3)
**S1—Sn1—S2**	114.444 (12)	**N2—C5—H5A**	107.5 (15)
**S1—Sn1—S3**^{i}	110.418 (13)	**N2—C5—H5B**	113.0 (16)
**S1—Sn1—S3**	110.437 (12)	**C4—C5—H5A**	109.6 (15)
**S2—Sn1—S3**^{i}	113.842 (13)	**C4—C5—H5B**	109.1 (16)
**S2—Sn1—S3**	113.728 (13)	**H5A—C5—H5B**	106 (2)
**S3—Sn1—S3**^{i}	91.856 (12)	**N2—C6—H6A**	110.2 (15)
**Sn1—S3—Sn1**^{i}	88.144 (12)	**N2—C6—C7**	110.85 (12)
**H1A—N1—H1B**	106 (2)	**N2—C6—H6B**	109.7 (15)
**H1A—N1—H1C**	110 (2)	**H6A—C6—H6B**	105 (2)
**H1B—N1—H1C**	108 (2)	**C7—C6—H6A**	110.0 (15)
**C4—N1—H1A**	109.6 (17)	**C7—C6—H6B**	110.4 (15)
**C4—N1—H1B**	111.4 (16)	**N2—C8—H8A**	109.9 (14)
**C4—N1—H1C**	111.9 (16)	**N2—C8—C9**	110.43 (13)
**C5—N2—C8**	110.81 (11)	**N2—C8—H8B**	111.5 (15)
**C6—N2—C5**	110.28 (11)	**H8A—C8—H8B**	111 (2)
**C6—N2—C8**	108.54 (11)	**C9—C8—H8A**	105.9 (14)
**H3A—N3—H3B**	113 (2)	**C9—C8—H8B**	108.4 (15)
**C7—N3—H3A**	106.6 (17)	**N3—C7—C6**	109.24 (12)
**C7—N3—H3B**	111.6 (14)	**N3—C7—H7A**	105.4 (16)
**C7—N3—C9**	110.54 (12)	**N3—C7—H7B**	111.4 (18)
**C9—N3—H3A**	111.1 (17)	**C6—C7—H7A**	110.0 (15)
**C9—N3—H3B**	104.2 (14)	**C6—C7—H7B**	111.2 (18)
**N1—C4—C5**	110.22 (11)	**H7A—C7—H7B**	109 (2)
**N1—C4—H4A**	108.3 (15)	**N3—C9—C8**	110.07 (12)
**N1—C4—H4B**	107.9 (15)	**N3—C9—H9A**	107.3 (17)
**C5—C4—H4A**	112.4 (15)	**N3—C9—H9B**	109.6 (15)
**C5—C4—H4B**	110.4 (15)	**C8—C9—H9A**	108.6 (17)
**H4A—C4—H4B**	108 (2)	**C8—C9—H9B**	107.6 (15)
**N2—C5—C4**	111.07 (11)	**H9A—C9—H9B**	114 (2)
**N1—C4—C5—N2**	−67.73 (15)	**C6—N2—C8—C9**	−60.84 (16)
**N2—C6—C7—N3**	−59.84 (16)	**C8—N2—C5—C4**	−83.90 (14)
**N2—C8—C9—N3**	58.16 (19)	**C8—N2—C6—C7**	62.04 (15)
**C5—N2—C6—C7**	−176.40 (12)	**C7—N3—C9—C8**	−55.62 (19)
**C5—N2—C8—C9**	177.92 (12)	**C9—N3—C7—C6**	56.02 (16)
**C6—N2—C5—C4**	155.90 (12)		

Symmetry code(s): (i) $-x+1, -y+2, -z+2$.

2.6 Hirshfeld surface of $AEPzH_2^{2+}$ molecular cations in $Sn_2S_6(AEPzH_2)_2$

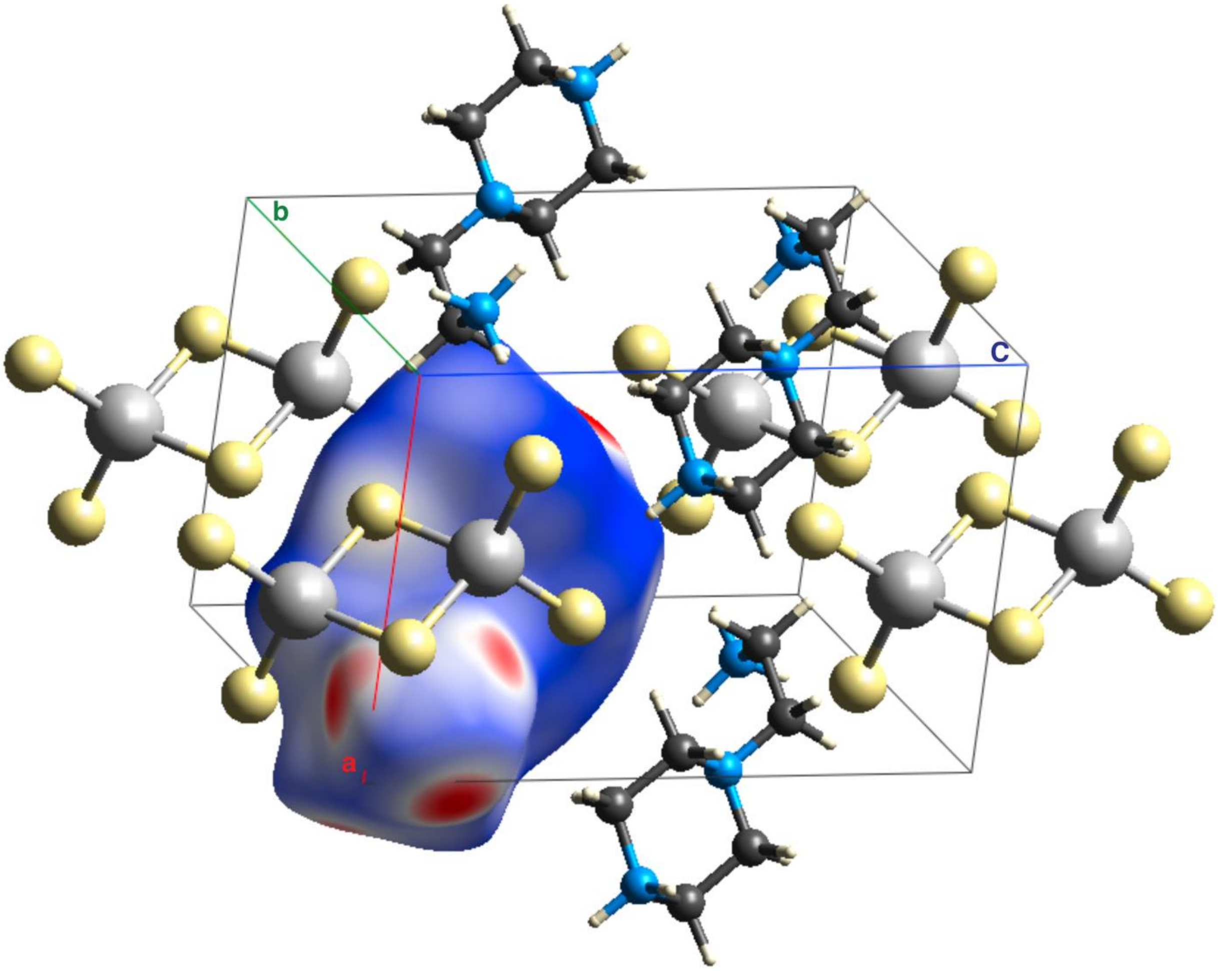

Fig. S9: Hirshfeld surface of an $AEPzH_2^{2+}$ cation in $Sn_2S_6(AEPzH_2)_2$. Colours on the surface represent distances between the surface and surrounding atoms, where the shortest contacts are shown in red, and the longest contacts are shown in blue. Colour scheme: C (black), H (white), N (blue), S (pale yellow), Sn (grey).

3. Compound: AEPz:EtOH-SnS-1

3.1 Optimizing crystal growth and collection of single crystal diffraction data

Several samples were synthesized at various conditions aiming to obtain crystals suitable for single crystal diffraction. The single crystal diffraction data were collected at room temperature due to crystal cracking upon cooling. A combined effect of using small crystals and collecting data at room temperature causes the single crystal data to be relatively weak. The crystal structure was solved in triclinic *P*-1. Structure solution was attempted in monoclinic *C*2/*m* (no. 12) without success.

In the refinements, a decent description of the organic species in-between the thiostannates layers was achieved by applying the EADP, SAME and DFIX restraints. By using the EADP restraint only, the AEPz molecules in the structure could be refined. However, the C-C bond lengths were varying widely in the range 1.4-1.9 Å. In order to overcome these issues, EADP and SAME restraints were applied to the two AEPz molecules (see also Fig. S12).

3.2 Low magnification SEM of AEPz:EtOH-SnS-1

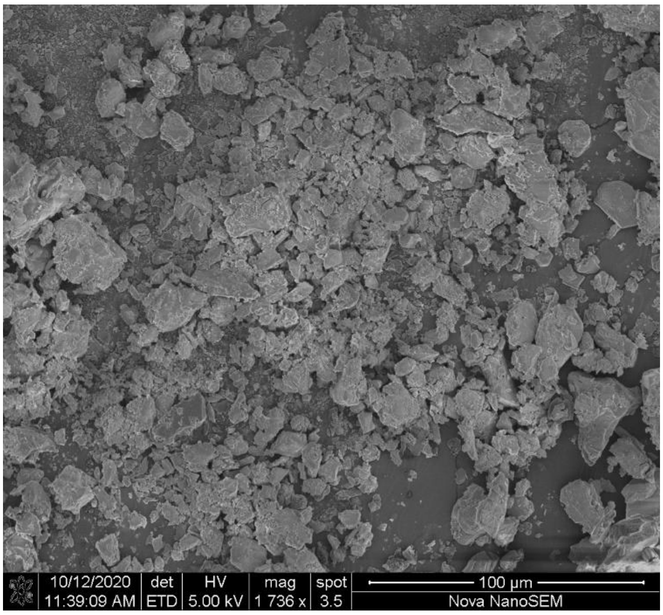

Fig. S10: Low magnification SEM of a sample of AEPz:EtOH-SnS-1.

3.3 Phase identification of AEPz:EtOH-SnS-1

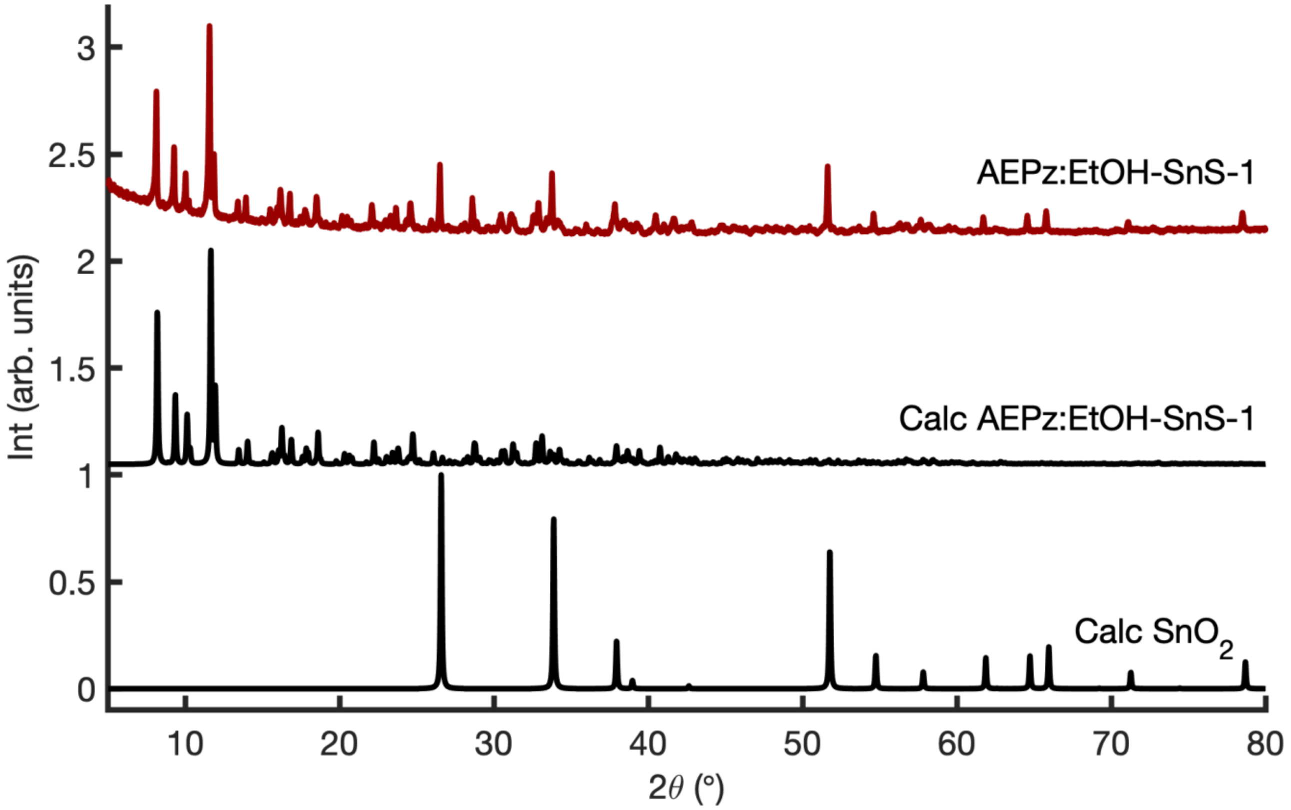

Fig. S11: Calculated patterns (black) of SnO_2 (ICSD entry 9163[2]) and AEPz:EtOH-SnS-1 based on the CIF data obtained in this work. Experimental PXRD data of AEPz:EtOH-SnS-1 (red) used in the Rietveld refinement (as presented in the manuscript).

3.4 AEPz:EtOH-SnS-1 crystal structure

AEPz molecule 1: The SAME command was restraining Part *a* [C5A-C10A, N3A-N5A] to Part *b* [C5B-C10B, N3B-N5B]. AEPz molecule 2: Part *a* [C11A-C16A, N6A-N8A] was likewise restrained to Part *b* [C11B-C16B, N6B-N8B]. In Part *a*, all C-N bonds were restrained through DFIX 1.47 (Å), and C-C were restrained to DFIX 1.54 (Å) to mimic real bond lengths. For every two AEPz molecules, one piperazine is present, for which the structure was also restrained using the EADP and DFIX commands. The data quality did not allow the hydrogen atoms to be observed in the electron density maps.

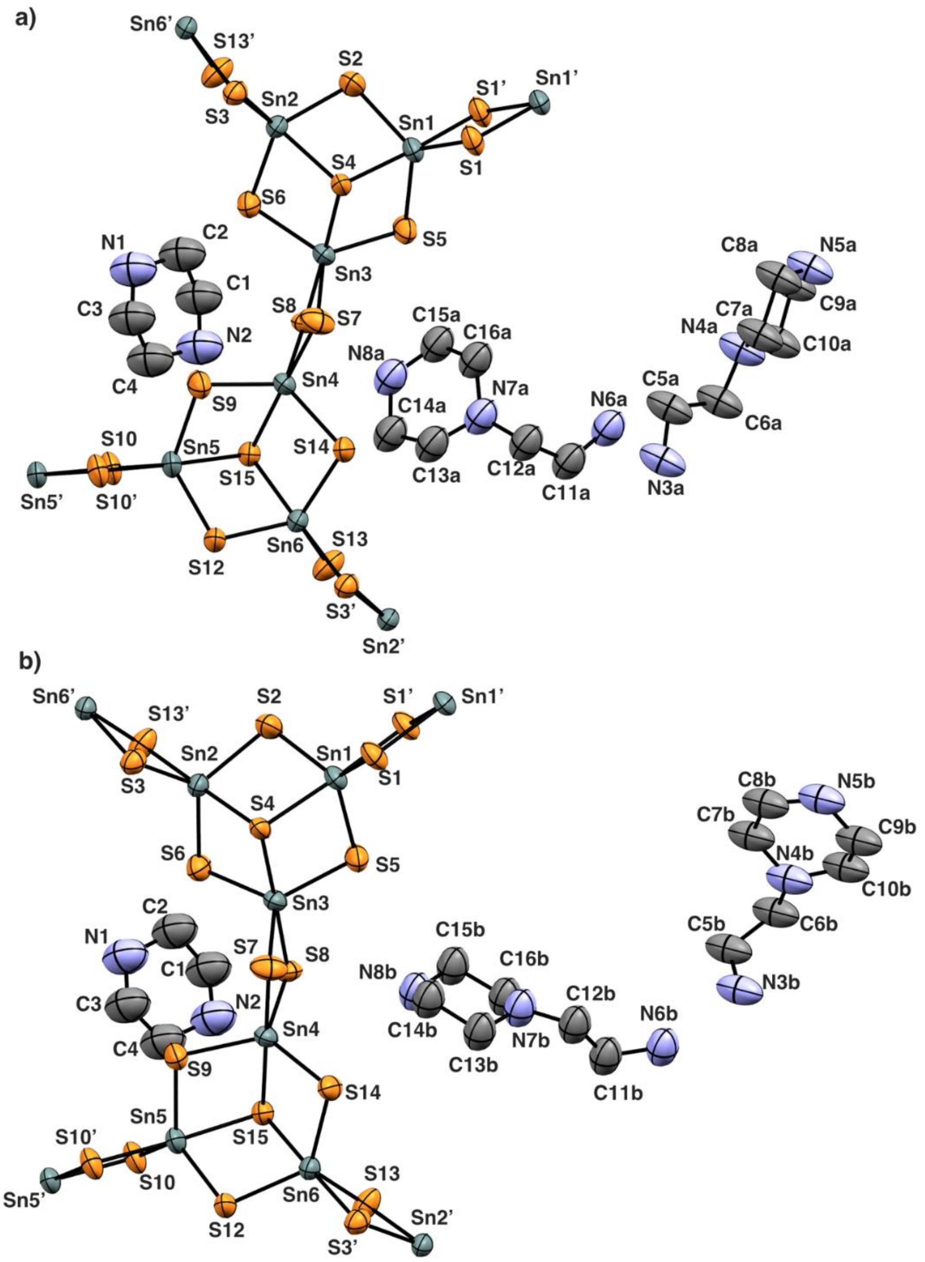

Fig. S12: ORTEP representation (50% probability ellipsoids) of a) Part *a* and b) Part *b* in AEPz:EtOH-SnS-1 with all atomic labels shown. Tin is shown in teal, sulfur in orange, carbon in grey, and nitrogen in blue. Hydrogen atoms have been omitted for clarity.

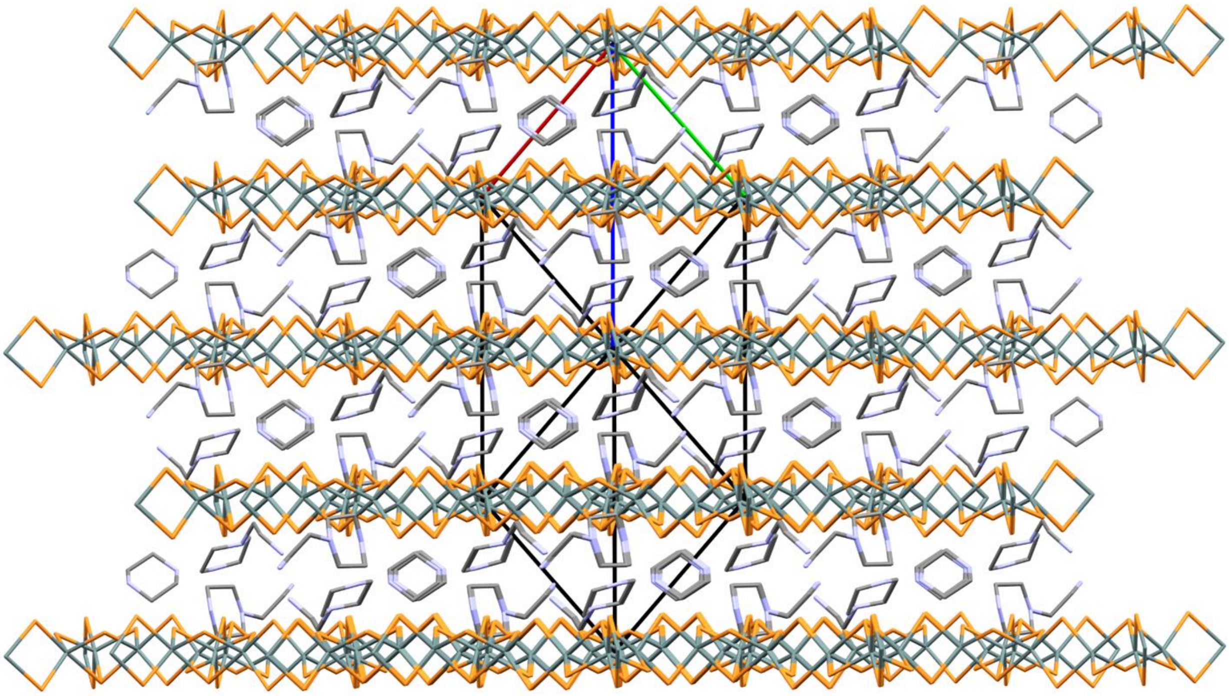

Fig. S13: Side view of AEPz-SnS-1 showing ordered organic molecular species (Part *b* only). Tin is shown in teal, sulfur in orange, carbon in grey, and nitrogen in blue. Hydrogen atoms have been omitted for clarity.

Table S6: Short contacts between nitrogen and sulfur atoms in the thiostannate layers of AEPz:EtOH-SnS-1. The primary and secondary amines are denoted by (1°) and (2°), respectively. The atomic labels refer to Fig. 5c, and the prime denotes a symmetry generated atom.

	**-S···N-**
**(2°) -N1···S14-**	3.90(3) Å
**(2°) -N2···S1'-**	3.40(3) Å
**(2°) -N5a···S3'-**	3.36(3) Å
**(2°) -N8a···S8-**	3.16(3) Å
**(1°) -N3a···S7'-**	3.49(6) Å
**(1°) -N6a···S13'-**	3.67(6) Å
**(2°) -N5b···S3'-**	3.17(4) Å
**(2°) -N8b···S8-**	3.33(3) Å
**(1°) -N3b···S7'-**	3.49(6) Å
**(1°) -N6b···S4'-**	3.44(5) Å

Table S7: Selected bond lengths, angles and torsion angles of AEPz:EtOH-SnS-1 (Å, º). The table has been generated using PublCIF[5].

**Sn3—S4**	2.589 (6)	**C7A—C8A**	1.57 (3)
**Sn3—S8**	2.576 (6)	**C6A—C5A**	1.55 (3)
**Sn3—S5**	2.425 (6)	**C5A—N3A**	1.458 (19)
**Sn3—S7**	2.362 (6)	**N4B—C10B**	1.48 (2)
**Sn3—S6**	2.425 (5)	**N4B—C7B**	1.49 (3)
**Sn6—S15**	2.587 (5)	**N4B—C6B**	1.48 (3)
**Sn6—S3^{i}**	2.574 (5)	**C10B—C9B**	1.536 (19)
**Sn6—S14**	2.411 (6)	**C9B—N5B**	1.47 (3)
**Sn6—S12**	2.440 (6)	**N5B—C8B**	1.46 (2)
**Sn6—S13**	2.362 (7)	**C7B—C8B**	1.555 (19)
**Sn4—S15**	2.611 (6)	**C6B—C5B**	1.54 (2)
**Sn4—S8**	2.419 (6)	**C5B—N3B**	1.45 (3)
**Sn4—S14**	2.428 (5)	**N7B—C12B**	1.54 (4)
**Sn4—S9**	2.422 (6)	**N7B—C13B**	1.47 (3)
**Sn4—S7**	2.488 (7)	**N7B—C16B**	1.46 (2)
**Sn2—S4**	2.606 (5)	**C12B—C11B**	1.54 (3)
**Sn2—S3**	2.399 (6)	**C11B—N6B**	1.50 (3)
**Sn2—S2**	2.416 (6)	**C13B—C14B**	1.56 (3)
**Sn2—S13^{ii}**	2.519 (6)	**C14B—N8B**	1.51 (3)
**Sn2—S6**	2.428 (7)	**N8B—C15B**	1.51 (2)
**Sn1—S4**	2.596 (6)	**C16B—C15B**	1.55 (3)
**Sn1—S5**	2.442 (7)	**N7A—C12A**	1.56 (4)
**Sn1—S1**	2.381 (6)	**N7A—C13A**	1.468 (19)
**Sn1—S1^{iii}**	2.553 (6)	**N7A—C16A**	1.480 (19)
**Sn1—S2**	2.415 (6)	**C12A—C11A**	1.543 (19)
**Sn5—S15**	2.596 (6)	**C11A—N6A**	1.479 (19)
**Sn5—S12**	2.432 (6)	**C13A—C14A**	1.546 (19)
**Sn5—S10^{iv}**	2.542 (6)	**C14A—N8A**	1.489 (19)
**Sn5—S10**	2.376 (6)	**N8A—C15A**	1.484 (18)
**Sn5—S9**	2.412 (7)	**C16A—C15A**	1.558 (19)
**N4A—C10A**	1.480 (19)	**N2—C1**	1.477 (18)
**N4A—C7A**	1.486 (19)	**N2—C4**	1.467 (18)
**N4A—C6A**	1.479 (19)	**C1—C2**	1.516 (19)
**C10A—C9A**	1.54 (3)	**N1—C3**	1.480 (18)
**C9A—N5A**	1.475 (19)	**N1—C2**	1.467 (18)
**N5A—C8A**	1.473 (19)	**C3—C4**	1.528 (19)
**S8—Sn3—S4**	178.57 (17)	**Sn5—S15—Sn4**	87.09 (19)
**S5—Sn3—S4**	88.0 (2)	**Sn3—S4—Sn2**	86.59 (18)
**S5—Sn3—S8**	92.5 (2)	**Sn3—S4—Sn1**	87.01 (19)
**S7—Sn3—S4**	92.7 (2)	**Sn1—S4—Sn2**	87.19 (17)
**S7—Sn3—S8**	88.0 (2)	**Sn4—S8—Sn3**	89.1 (2)
**S7—Sn3—S5**	123.2 (2)	**Sn2—S3—Sn6^{ii}**	89.5 (2)
**S7—Sn3—S6**	121.4 (2)	**Sn6—S14—Sn4**	94.83 (19)
**S6—Sn3—S4**	87.51 (19)	**Sn3—S5—Sn1**	94.3 (2)
**S6—Sn3—S8**	91.1 (2)	**Sn5—S12—Sn6**	94.3 (2)
**S6—Sn3—S5**	115.3 (2)	**Sn1—S1—Sn1^{iii}**	90.3 (2)
**S3^{i}—Sn6—S15**	178.89 (19)	**Sn5—S10—Sn5^{iv}**	90.6 (2)
**S14—Sn6—S15**	87.63 (19)	**Sn1—S2—Sn2**	95.9 (2)
**S14—Sn6—S3^{i}**	91.3 (2)	**Sn6—S13—Sn2^{i}**	91.7 (2)
**S14—Sn6—S12**	114.7 (2)	**Sn5—S9—Sn4**	95.8 (2)
**S12—Sn6—S15**	87.81 (18)	**Sn3—S7—Sn4**	92.6 (2)
**S12—Sn6—S3^{i}**	92.84 (19)	**C10A—N4A—C7A**	119 (3)
**S13—Sn6—S15**	92.0 (2)	**C6A—N4A—C10A**	110 (3)

**S13—Sn6—S3^{i}**	88.4 (2)	**C6A—N4A—C7A**	112 (3)
**S13—Sn6—S14**	122.0 (2)	**N4A—C10A—C9A**	107 (3)
**S13—Sn6—S12**	123.3 (2)	**N5A—C9A—C10A**	111 (3)
**S8—Sn4—S15**	94.62 (19)	**C8A—N5A—C9A**	122 (3)
**S8—Sn4—S14**	124.1 (2)	**N4A—C7A—C8A**	110 (3)
**S8—Sn4—S9**	119.9 (2)	**N5A—C8A—C7A**	109 (3)
**S8—Sn4—S7**	88.9 (2)	**N4A—C6A—C5A**	114 (3)
**S14—Sn4—S15**	86.76 (19)	**N3A—C5A—C6A**	111 (3)
**S14—Sn4—S7**	91.1 (2)	**C10B—N4B—C7B**	119 (3)
**S9—Sn4—S15**	86.4 (2)	**C10B—N4B—C6B**	111 (3)
**S9—Sn4—S14**	116.0 (2)	**C6B—N4B—C7B**	112 (3)
**S9—Sn4—S7**	92.2 (2)	**N4B—C10B—C9B**	108 (3)
**S7—Sn4—S15**	176.5 (2)	**N5B—C9B—C10B**	111 (3)
**S3—Sn2—S4**	94.74 (19)	**C8B—N5B—C9B**	124 (3)
**S3—Sn2—S2**	120.0 (2)	**N4B—C7B—C8B**	111 (3)
**S3—Sn2—S13^{ii}**	88.9 (2)	**N5B—C8B—C7B**	111 (3)
**S3—Sn2—S6**	123.8 (2)	**N4B—C6B—C5B**	115 (3)
**S2—Sn2—S4**	86.42 (18)	**N3B—C5B—C6B**	113 (3)
**S2—Sn2—S13^{ii}**	91.9 (2)	**C13B—N7B—C12B**	110 (3)
**S2—Sn2—S6**	116.2 (2)	**C16B—N7B—C12B**	127 (3)
**S13^{ii}—Sn2—S4**	176.4 (2)	**C16B—N7B—C13B**	115 (3)
**S6—Sn2—S4**	87.05 (19)	**C11B—C12B—N7B**	113 (3)
**S6—Sn2—S13^{ii}**	90.8 (2)	**N6B—C11B—C12B**	122 (3)
**S5—Sn1—S4**	87.5 (2)	**N7B—C13B—C14B**	113 (3)
**S5—Sn1—S1^{iii}**	93.3 (2)	**N8B—C14B—C13B**	104 (3)
**S1^{iii}—Sn1—S4**	176.62 (18)	**C15B—N8B—C14B**	122 (3)
**S1—Sn1—S4**	92.62 (19)	**N7B—C16B—C15B**	116 (3)
**S1—Sn1—S5**	122.8 (2)	**N8B—C15B—C16B**	105 (3)
**S1—Sn1—S1^{iii}**	89.7 (2)	**C13A—N7A—C12A**	111 (3)
**S1—Sn1—S2**	123.7 (2)	**C13A—N7A—C16A**	114 (3)
**S2—Sn1—S4**	86.66 (19)	**C16A—N7A—C12A**	124 (3)
**S2—Sn1—S5**	113.5 (2)	**C11A—C12A—N7A**	111 (3)
**S2—Sn1—S1^{iii}**	90.0 (2)	**N6A—C11A—C12A**	126 (3)
**S12—Sn5—S15**	87.81 (18)	**N7A—C13A—C14A**	115 (3)
**S12—Sn5—S10^{iv}**	92.7 (2)	**N8A—C14A—C13A**	106 (3)
**S10—Sn5—S15**	92.90 (19)	**C15A—N8A—C14A**	126 (3)
**S10^{iv}—Sn5—S15**	176.9 (2)	**N7A—C16A—C15A**	113 (3)
**S10—Sn5—S12**	123.4 (2)	**N8A—C15A—C16A**	106 (3)
**S10—Sn5—S10^{iv}**	89.4 (2)	**Sn3—S6—Sn2**	94.5 (2)
**S10—Sn5—S9**	123.2 (2)	**C4—N2—C1**	113 (2)
**S9—Sn5—S15**	86.9 (2)	**N2—C1—C2**	110 (3)
**S9—Sn5—S12**	113.4 (2)	**C2—N1—C3**	118 (3)
**S9—Sn5—S10^{iv}**	90.1 (2)	**N1—C3—C4**	105 (2)
**Sn6—S15—Sn4**	86.54 (17)	**N1—C2—C1**	109 (3)
**Sn6—S15—Sn5**	87.10 (17)	**N2—C4—C3**	114 (3)

**N4A—C10A—C9A—N5A**	49 (5)	**C12B—N7B—C13B—C14B**	154 (4)
**N4A—C7A—C8A—N5A**	−43 (6)	**C12B—N7B—C16B—C15B**	−163 (4)
**N4A—C6A—C5A—N3A**	−153 (4)	**C13B—N7B—C12B—C11B**	118 (4)
**C10A—N4A—C7A—C8A**	55 (5)	**C13B—N7B—C16B—C15B**	50 (5)
**C10A—N4A—C6A—C5A**	−160 (4)	**C13B—C14B—N8B—C15B**	−58 (5)
**C10A—C9A—N5A—C8A**	−51 (6)	**C14B—N8B—C15B—C16B**	53 (5)
**C9A—N5A—C8A—C7A**	47 (6)	**C16B—N7B—C12B—C11B**	−31 (7)
**C7A—N4A—C10A—C9A**	−57 (5)	**C16B—N7B—C13B—C14B**	−54 (5)
**C7A—N4A—C6A—C5A**	65 (5)	**N7A—C12A—C11A—N6A**	78 (6)
**C6A—N4A—C10A—C9A**	172 (4)	**N7A—C13A—C14A—N8A**	−44 (5)

**C6A—N4A—C7A—C8A**	−175 (4)	**N7A—C16A—C15A—N8A**	47 (5)
**N4B—C10B—C9B—N5B**	47 (5)	**C12A—N7A—C13A—C14A**	−160 (3)
**N4B—C7B—C8B—N5B**	−36 (6)	**C12A—N7A—C16A—C15A**	164 (3)
**N4B—C6B—C5B—N3B**	69 (6)	**C13A—N7A—C12A—C11A**	119 (4)
**C10B—N4B—C7B—C8B**	52 (5)	**C13A—N7A—C16A—C15A**	−55 (5)
**C10B—N4B—C6B—C5B**	−151 (4)	**C13A—C14A—N8A—C15A**	46 (6)
**C10B—C9B—N5B—C8B**	−43 (6)	**C14A—N8A—C15A—C16A**	−48 (6)
**C9B—N5B—C8B—C7B**	37 (7)	**C16A—N7A—C12A—C11A**	−99 (4)
**C7B—N4B—C10B—C9B**	−57 (5)	**C16A—N7A—C13A—C14A**	54 (5)
**C7B—N4B—C6B—C5B**	74 (5)	**N2—C1—C2—N1**	−53 (4)
**C6B—N4B—C10B—C9B**	171 (3)	**C1—N2—C4—C3**	−57 (4)
**C6B—N4B—C7B—C8B**	−177 (4)	**N1—C3—C4—N2**	52 (4)
**N7B—C12B—C11B—N6B**	138 (4)	**C3—N1—C2—C1**	58 (4)
**N7B—C13B—C14B—N8B**	53 (5)	**C2—N1—C3—C4**	−55 (4)
**N7B—C16B—C15B—N8B**	−45 (6)	**C4—N2—C1—C2**	55 (4)

Symmetry codes: (i) *x*+1, *y*−1, *z*; (ii) *x*−1, *y*+1, *z*; (iii) −*x*−1, −*y*+1, −*z*+1; (iv) −*x*+1, −*y*+1, −*z*.

3.5 Rietveld refinements of AEPz:EtOH-SnS-1

Rietveld refinement of PXRD data collected on AEPz:EtOH-SnS-1 was performed in order to estimate the SnO_2 content in the sample.

CIF data for AEPz:EtOH-SnS-1 and SnO_2 (ICSD entry 9163[2]) were used as starting models and all atomic coordinates were fixed throughout the refinement. The absorption was estimated to be μR = 2.9. The peak profiles were described using a Thompson-Cox-Hastings pseudo-Voigt function with axial divergence asymmetry, and only X and W were refined, whereas U and V were restrained to 0.00413 and -0.00762, respectively. Both phases were refined using an overall B-factor, and asymmetry was described by the Asym1 and Asym2 parameters.

The resulting fit is shown in Fig. S14, and the refined parameters are summarised in Table S8. Based on the refinement, the SnO_2 impurity is estimated to be approximately 11% by mass.

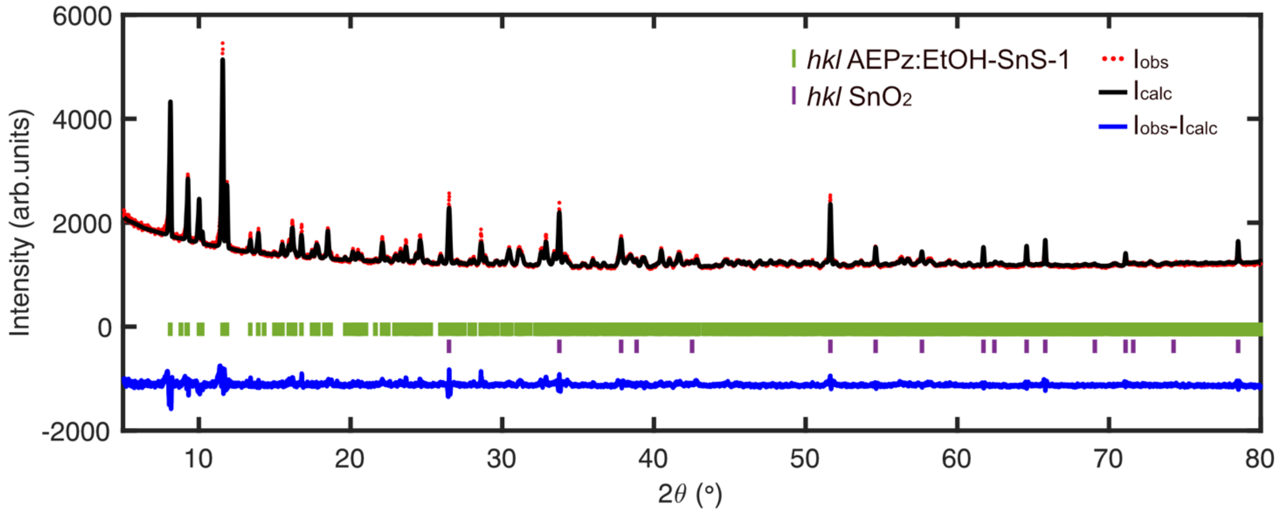

Fig. S14: Rietveld refinement of PXRD data collected on a sample of AEPz:EtOH-SnS-1 with an SnO_2 impurity. A manual selection of background points was used in the refinement. The refinement is displayed as measured intensities (red dots), calculated pattern (black), difference curve I_{obs}-I_{calc} (blue), Bragg positions for AEPz:EtOH-SnS-1 (green) and SnO_2 (purple).

Table S8: Refined parameters for AEPz:EtOH-SnS-1 and SnO_2. Numbers marked by an asterisk (*) denote non-zero parameters that were not refined.

	SnO₂	*AEPz:EtOH-SnS-1*
Bragg R-factor (%)	5.48	17.8
R_F-factor (%)	2.92	16.7
Fraction (%)	10.9(1)	89.1(5)
	**Refined parameters**	
Zero	-0.0572(5)	
Scale factor	$1.785(22)\cdot10^{-2}$	$4.699(18)\cdot10^{-3}$
a (Å)	4.74519(6)	11.86212(48)
b (Å)	-	11.89902(48)
c (Å)	3.19192(6)	18.32413(54)
α (°)	-	76.2992(36)
β (°)	-	76.3468(36)
γ (°)	-	67.2765(24)
U	0.00413*	0.00413*
V	-0.00762*	-0.00762*
W	0.0389(3)	0.01351(19)
X	0.0189*	0.2211(87)
Asym1	-	0.0837(32)
Asym2	-	0.03770(88)
Overall *B*-factor ($Å^2$)	0.685(92)	1.161(61)

4. Diffuse reflectance spectroscopy of pristine compounds

Band gaps of the pristine materials were determined by transforming diffuse reflectance spectroscopy (DRS) data by the Kubelka-Munk method. The raw DRS data are shown in Fig. S15, while the Kulbelka-Munk transformed data (shown in the manuscript) were used for band gap determination. In order to determine the band gap, a background was fitted to the part of the spectrum with lower photon energy than the absorption edge, and a tangent line was fitted to the sloped region of the curve. The bandgap was determined from the intersection of these lines as shown in Fig. S16.

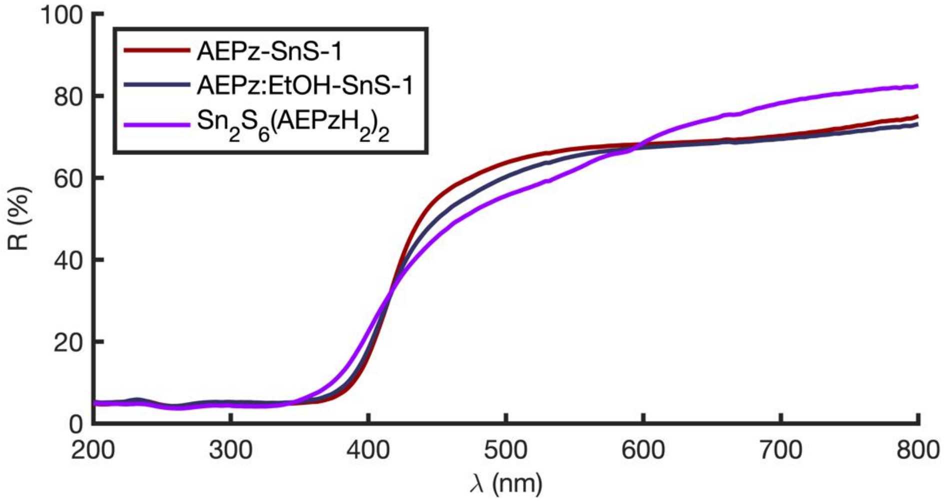

Fig. S15: DRS data of the pristine samples of AEPz-SnS1 (red), AEPz:EtOH-SnS-1 (navy blue) and $Sn_2S_6(AEPzH_2)_2$ (purple) all showing similar optical properties.

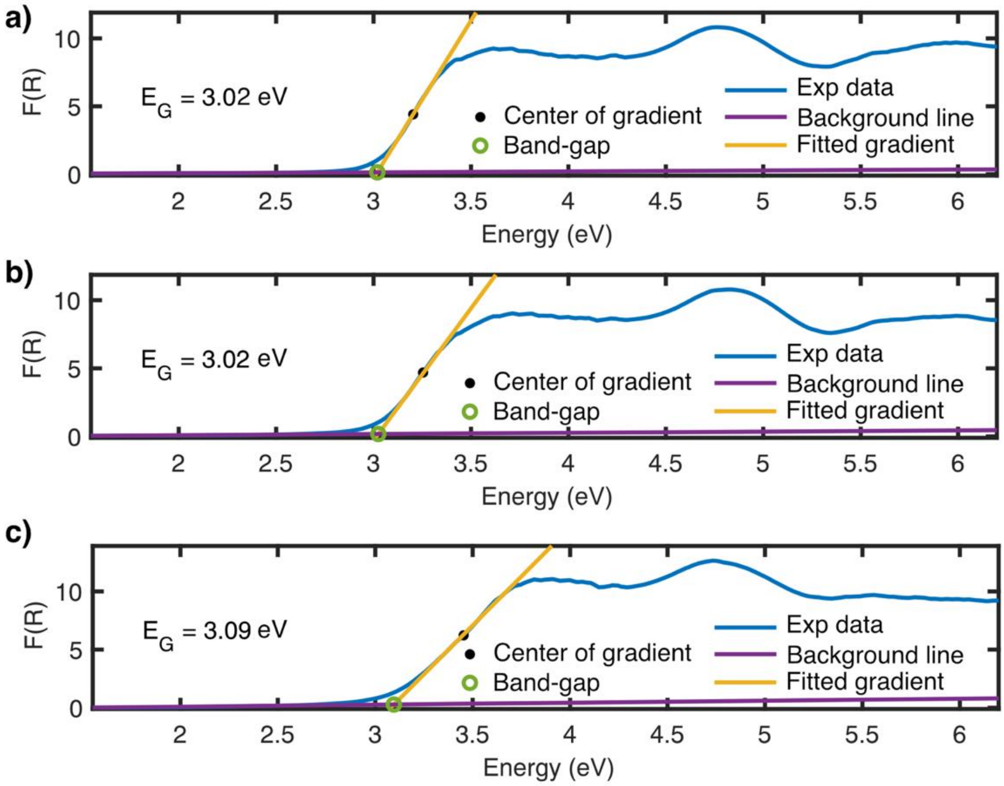

Fig. S16: Kubelka-Munk $F(R)$ functions fitted for band gap determination of pristine a) AEPz-SnS-1, b) AEPz:EtOH-SnS-1 and c) $Sn_2S_6(AEPzH_2)_2$. The blue lines are the transformed experimental data. The yellow and purple lines are the fitted tangent and background lines, respectively. The black dots are the centers of the tangent lines. The green circles are the intersections of the tangent and background lines and are indicating the band gap energy.

5. Stability studies of AEPz-SnS-1 and AEPz:EtOH-SnS-1

5.1 AEPz:EtOH-SnS-1 water treatment

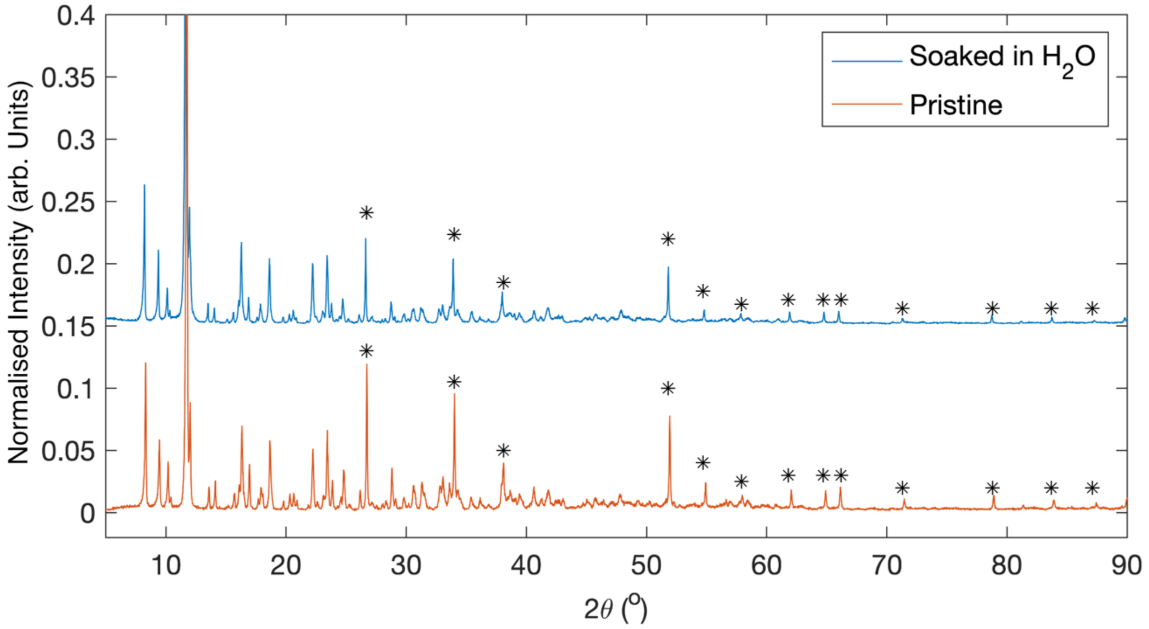

Fig. S17: PXRD of pristine AEPz:EtOH-SnS-1 (orange) and a sample of AEPz:EtOH-SnS-1 after soaking in water (blue). The asterisks (*) indicate the presence of an SnO_2 impurity. Intensities were normalised to the Bragg peak at 12°.

5.2 CHNS analysis of AEPz-SnS-1 and AEPz:EtOH-SnS-1

Table S9. CHNS analysis of pristine and heat treated AEPz-SnS-1 and AEPz:EtOH-SnS-1. The calculated values are based of CIF data and the assumption of two AEPz molecules per [$Sn_3S_7^{2-}$] in AEPz-SnS-1. Mass fractions are given in %.

	AEPz-SnS-1				*AEPz:EtOH-SnS-1*			
	**C**	**H**	**N**	**S**	**C**	**H**	**N**	**S**
Pristine Calc	17.14	3.84	9.99	26.69	12.70	2.94	7.38	29.73
Pristine	18.12(5)	4.44(2)	10.46(2)	24.16(10)	12.47(14)	3.04(1)	7.10(5)	25.17(12)
100 °C	17.98(4)	4.45(6)	10.40(2)	24.64(2)	12.28(5)	2.94(6)	7.01(3)	25.13(12)
150 °C	17.56(3)	4.18(9)	10.11(2)	25.02(5)	12.30(7)	2.90(7)	7.01(4)	25.33(11)
200 °C	17.20(3)	3.56(2)	9.34(2)	25.25(3)	12.04(8)	2.84(10)	6.77(4)	25.07(12)

5.3 AEPz:EtOH-SnS-1 thermal treatment

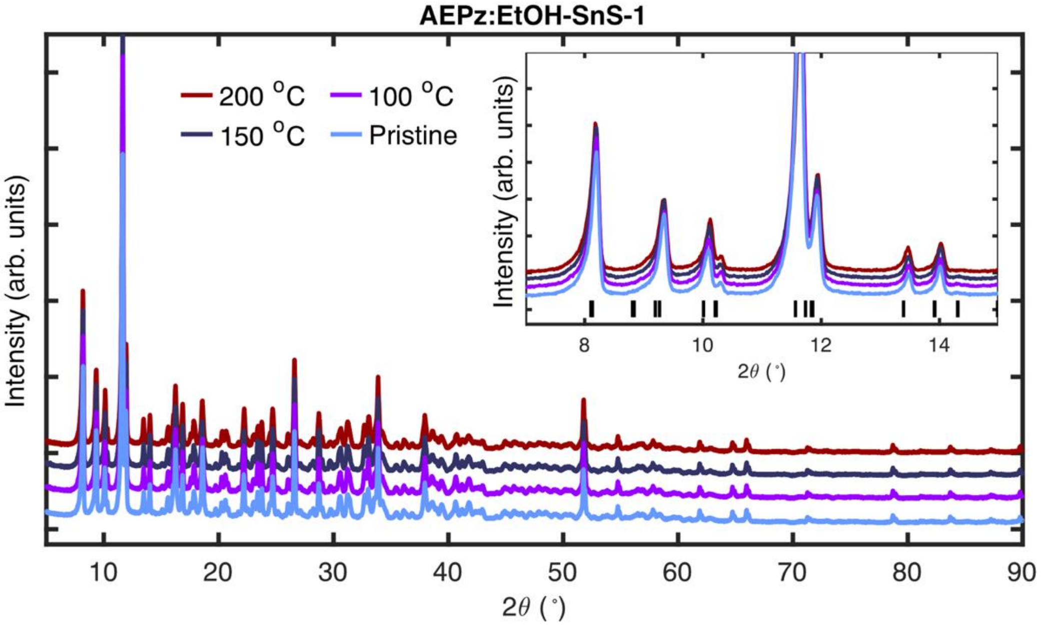

Fig. S18: PXRD of the heat treated samples of pristine AEPz:EtOH-SnS-1 (light blue) and samples heated to 100 ºC (purple), 150 ºC (navy blue) and 200 ºC (red). Data were measured on a Malvern-Panalytical Aeris diffractometer (Cu *K*α radiation) using a flat sample holder in Bragg-Brentano geometry. Each diffractogram is shifted along the ordinate for clarity.

5.4 Le Bail fits to PXRD data of heat treated AEPz-SnS-1

PXRD data of the three heat treated samples of AEPz-SnS-1 are shown in Fig. S19. The data clearly show new peaks at 10.3°, 13.9° and 19.4° after the heat treatment, but the overall structure remains the same. We speculate that the additional peaks are indicative of a lowering in crystal symmetry. The programme CellMuncher (part of the Dr. Probe package[6]) was used to describe a potential symmetry change from hexagonal to orthorhombic, but a new unit cell matching all Bragg peaks was not successfully obtained.

The PXRD data were refined by the Le Bail method by using CIF data for AEPz-SnS-1 as structural model. A Thompson-Cox-Hastings pseudo-Voigt function with axial divergence asymmetry was applied to describe the peak profiles. Manual background points were selected and fixed, and the unit cells of SnO_2 (ICSD entry 9163[2]) and AEPz-SnS-1 were refined in addition to the zero point. Only *W* was refined for the 100 °C data, whereas both *W* and *X* were refined to describe the peak profiles of the 150 °C and 200 °C data. *U* and *V* were fixed to 0.00413 and -0.00762, respectively, in all refinements. To describe asymmetry, the Asym1 and Asym2 parameters were refined. The refined plots are shown in Fig. S20, and the refined parameters are shown in Table S10.

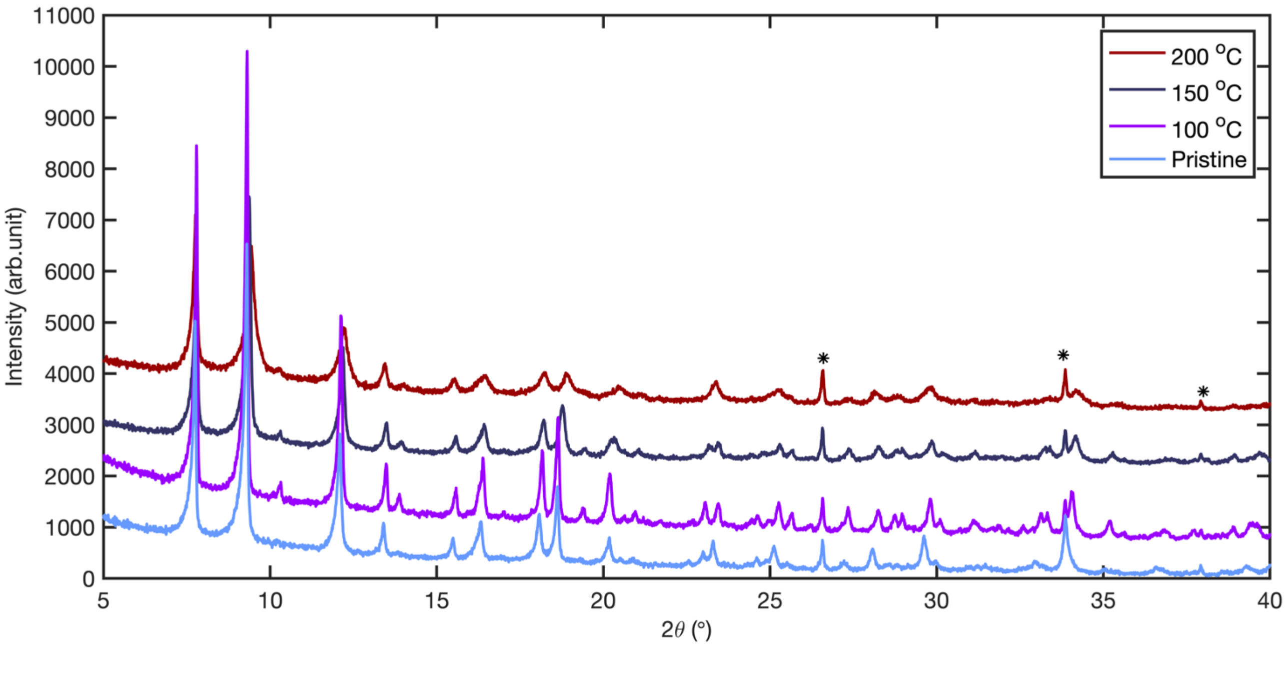

Fig. S19: PXRD of the heat treated samples of pristine AEPz-SnS-1 (light blue) and samples heated to 100 °C (purple), 150 °C (navy blue) and 200 °C (red). Peaks marked by asterisks (*) indicate the SnO_2 impurity.

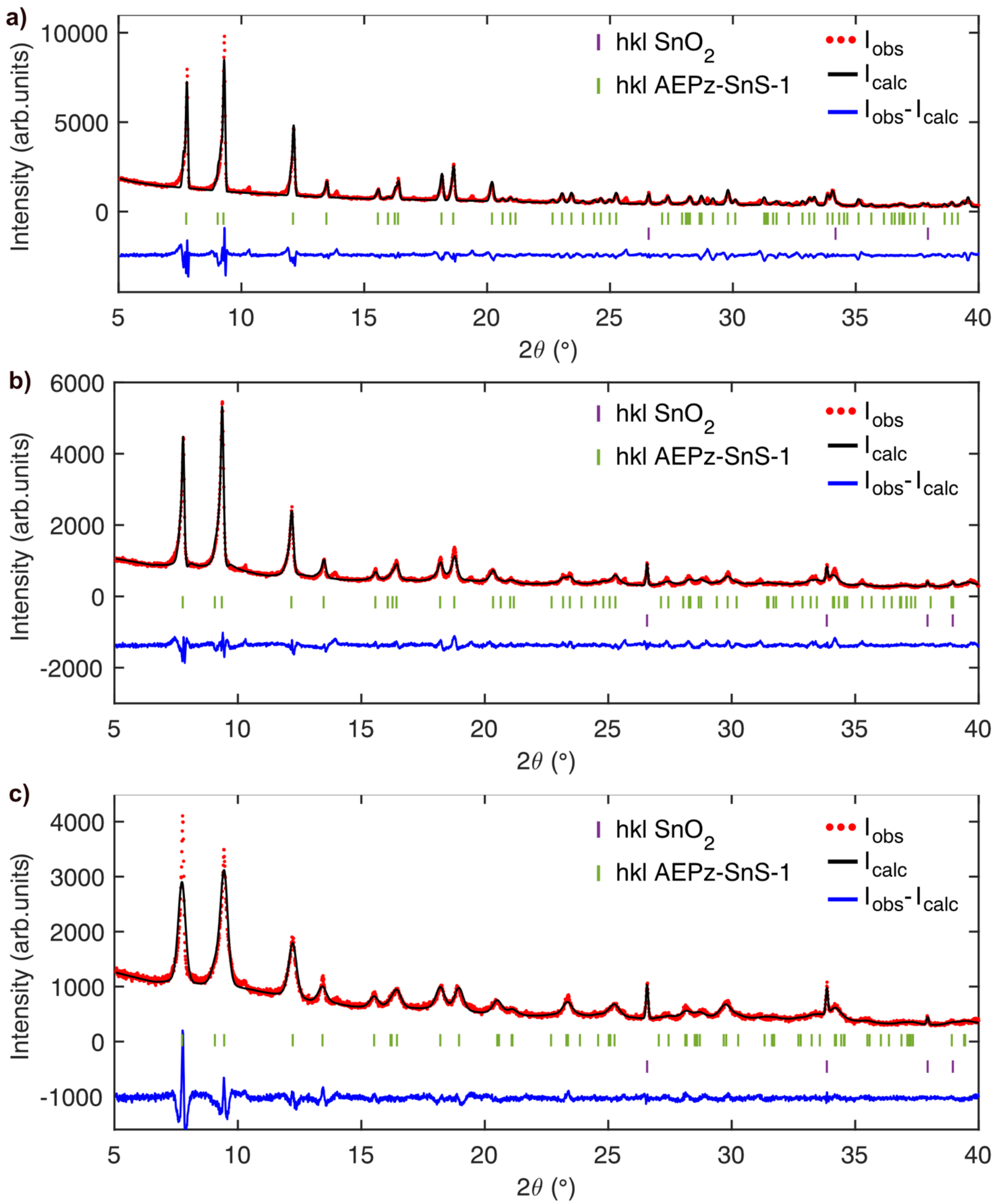

Fig. S20: Le Bail fits to samples of heat treated AEPz-SnS-1 using CIF data for AEPz-SnS-1 as starting model. Data were collected at room temperature after the heat treatment. a) 100 ºC, b) 150 ºC and c) 200 ºC. The refinements are displayed as measured intensities (red dots), calculated pattern (black), difference curve I_{obs}-I_{calc} (blue), Bragg positions for AEPz-SnS-1 (green) and SnO_2 (purple) are shown as vertical lines.

Table S10: Refined parameters from Le Bail Refinements of data as displayed in Fig. S20. Numbers marked by an asterisk (*) denote non-zero parameters that were not refined. Numbers marked by two asterisks (**) denote non-zero parameters that have been refined and then fixed.

	100ºC		***150 ºC***		***200 ºC***	
	**SnO_2**	**AEPz-SnS-1**	**SnO_2**	**AEPz-SnS-1**	**SnO_2**	**AEPz-SnS-1**
R_p (%)	44.6	10.3	25.5	6.31	27.6	5.44
R_{wp} (%)	23.8	12.5	14.5	7.64	26.5	9.69
	**Refined parameters**		**Refined parameters**		**Refined parameters**	
Zero	-0.0137(62)		0.0121**		-0.001(22)	
a (Å)	4.73786(73)	13.1341(14)	4.74029(16)	13.13986(86)	4.7397(19)	13.1761(53)
c (Å)	3.1451(15)	19.0344(24)	3.18876(21)	18.9107(10)	3.18850(93)	18.7101(82)
U	0.00413*	0.00413*	0.00413*	0.00413*	0.00413*	0.00413*
V	-0.00762*	-0.00762*	-0.00762*	-0.00762*	-0.00762*	-0.00762*
W	0.0170(27)	0.02657(25)	0.01032(58)	0.00266(26)	0.01278(92)	0.09222**
X	0.00*	0.00*	0.00*	1.70(13)	0.00	1.345(25)
Asym1	-	0.109(12)	-	0.0477(16)	-	0.017(13)
Asym2	-	0.0615(23)	-	0.02126(38)	-	0.0047(16)
Overall *B*-factor ($Å^2$)	1.00*	1.00*	1.00*	1.00*	1.00*	1.00*

5.5 Pair distribution function analysis of heat treated AEPz-SnS-1

Pair distribution functions (PDFs) of heat treated AEPz-SnS-1 samples are plotted as $G(r)$ in Fig. S21 and as distance weighted $G(r)\cdot r$ functions in Fig. S22. The calculated PDF of AEPz-SnS-1 is shown as $G(r)$ in all figures.

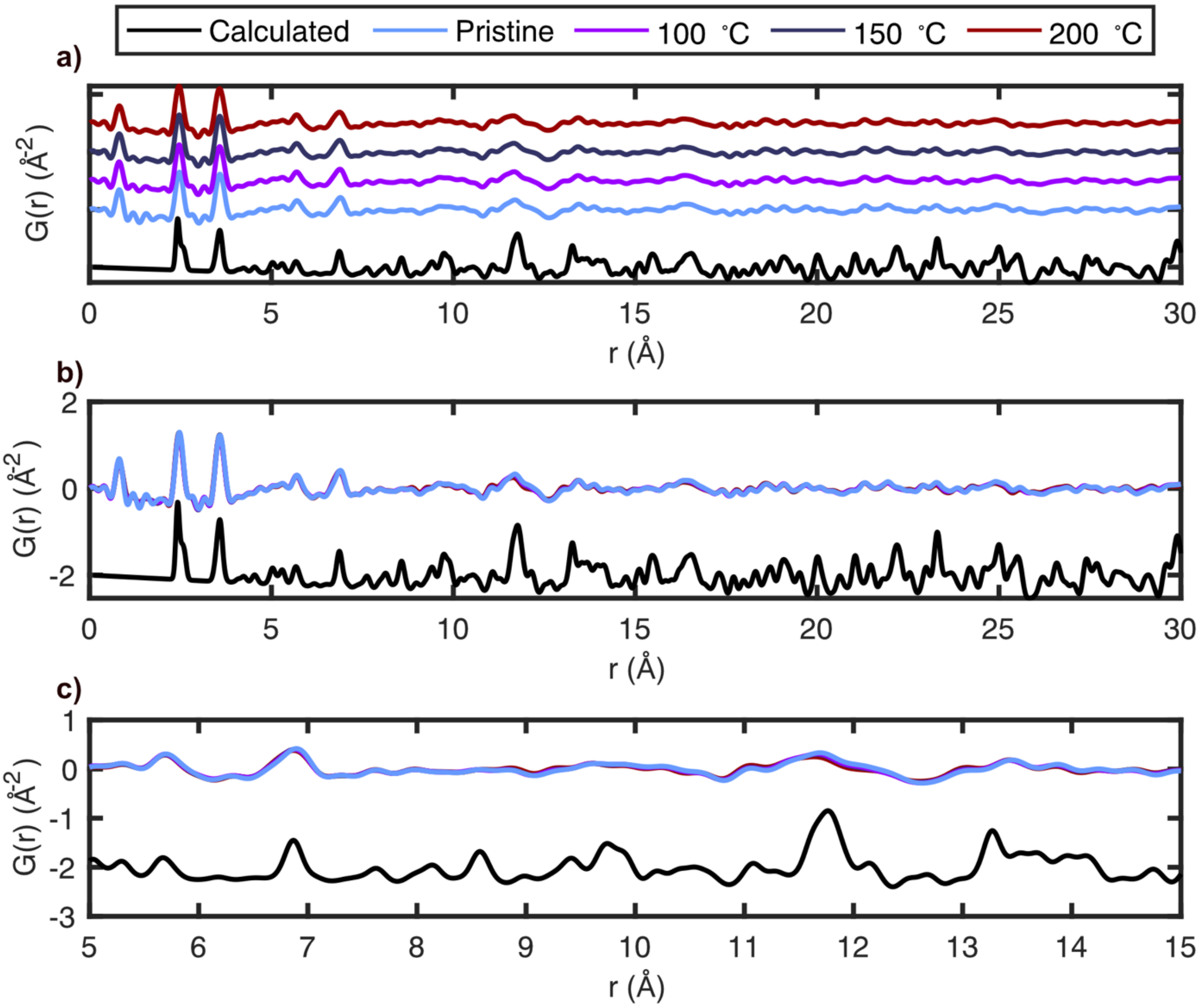

Fig. S21: PDFs of heat treated AEPz-SnS-1 powders shown as $G(r)$: calculated pattern (black), pristine (light blue), 100 ºC (purple), 150 ºC (navy blue), and 200 ºC (red). All calculated patterns have been shifted by -2 along the ordinate. a) Data for all samples displayed as individual PDFs (shifted along the ordinate for clarity), b) Superimposed data for all samples (r = 0-30 Å). c) Superimposed PDF data (r=5-15 Å).

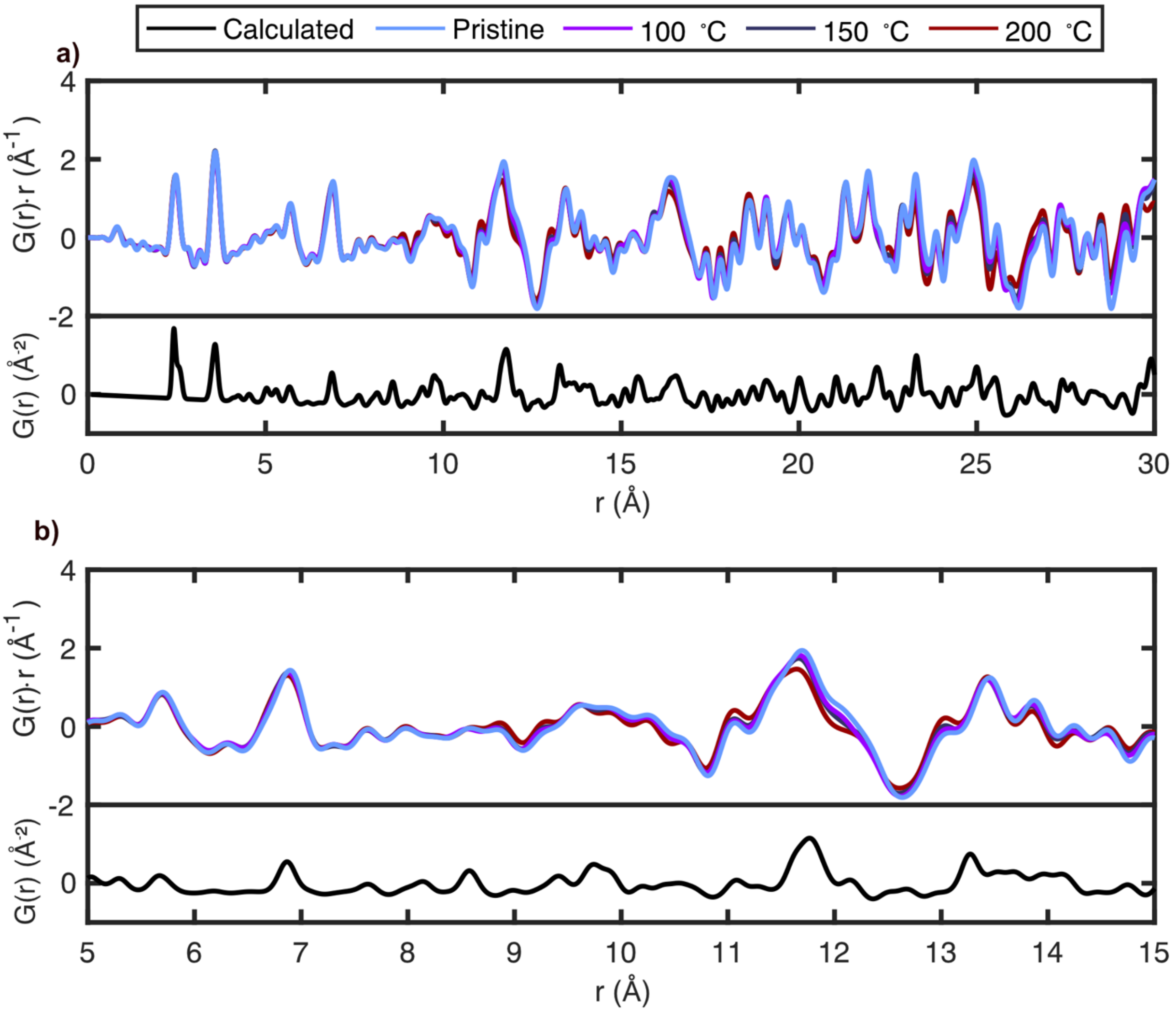

Fig. S22: PDF of heat treated AEPz-SnS-1 powders displayed as $G(r)\cdot r$: calculated pattern (black), pristine (light blue), 100 °C (purple), 150 °C (navy blue) and 200 °C (red). The calculated patterns are displayed as $G(r)$. a) Superimposed $G(r)\cdot r$ data for all samples (r = 0-30 Å). b) Superimposed $G(r)\cdot r$ data for all samples (r=5-15 Å).

5.6 Diffuse reflectance spectroscopy of heat treated samples

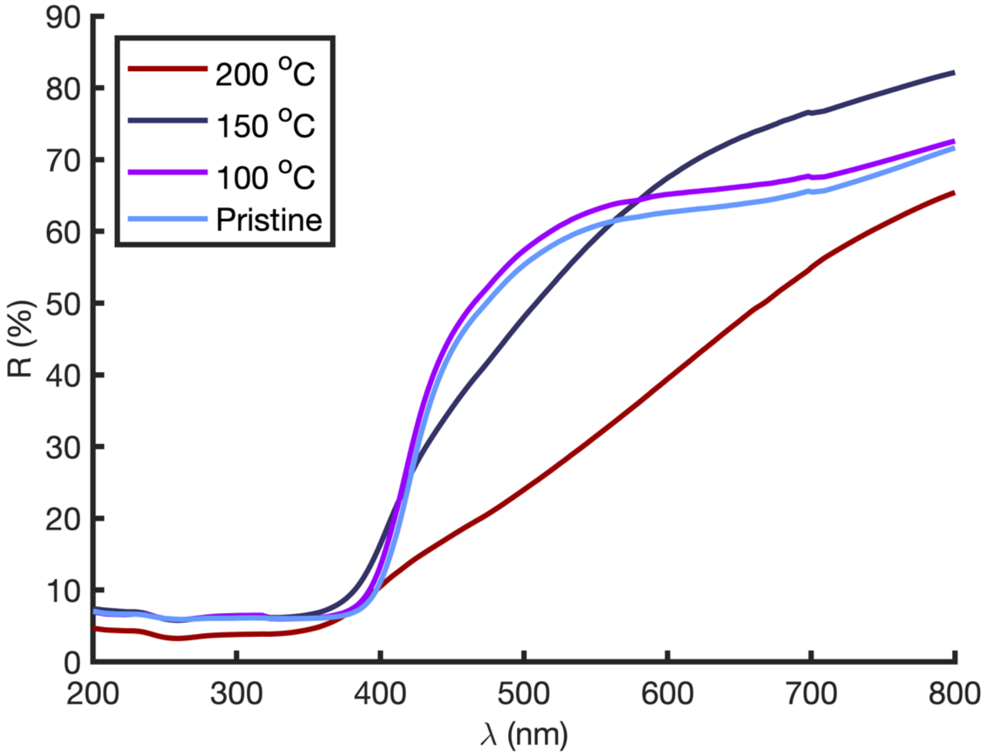

Fig. S23: Diffuse reflectance spectroscopy data of heat treated samples of AEPz-SnS-1. The pristine sample (light blue) and the 100 ºC sample (purple) are very similar, whereas for samples treated at 150 ºC (navy blue) and 200 ºC (red) the absorption curves shift into longer wavelengths, and the materials have a less defined band gap.

6. References

1 Hvid, M. S. *et al.* Structural changes during water-mediated amorphization of semiconducting two-dimensional thio-stannates. *IUCrJ* **6**, 804-814, doi:10.1107/S2052252519006791 (2019).

2 Baur, W. H. & Khan, A. A. Rutile-type compounds. IV. SiO2, GeO2 and a comparison with other rutile-type structures. *Acta Crystallogr. B* **27**, 2133-2139, doi:10.1107/s0567740871005466 (1971).

3 Filso, M. O., Chaaban, I., Al Shehabi, A., Skibsted, J. & Lock, N. The structure-directing amine changes everything: structures and optical properties of two-dimensional thiostannates. *Acta Crystallogr. B* **73**, 931-940, doi:10.1107/S2052520617010630 (2017).

4 Pienack, N. *et al.* New Thiostannates Synthesized Under Solvothermal Conditions: Crystal Structures of (trenH)2Sn3S7 and {[Mn(tren)]2Sn2S6}. *Z. Naturforsch.* **67**, 1098-1106, doi:10.5560/znb.2012-0126 (2012).

5 Westrip, S. P. publCIF: software for editing, validating and formatting crystallographic information files. *J. Appl. Crystallogr.* **43**, 920-925, doi:10.1107/s0021889810022120 (2010).

6 Barthel, J. Dr. Probe: A software for high-resolution STEM image simulation. *Ultramicroscopy* **193**, 1-11, doi:10.1016/j.ultramic.2018.06.003 (2018).
